# *In silico* Metabolic Pathway Analysis Identifying Target Against Leishmaniasis – A Kinetic Modeling Approach

**DOI:** 10.3389/fgene.2020.00179

**Published:** 2020-03-06

**Authors:** Nikita Bora, Anupam Nath Jha

**Affiliations:** Computational Biophysics Laboratory, Department of Molecular Biology and Biotechnology, Tezpur University, Tezpur, India

**Keywords:** *Leishmania donovani*, Visceral Leishmaniasis, kinetic modeling, purine salvage, protein protein interaction

## Abstract

The protozoan *Leishmania donovani*, from trypanosomatids family is a deadly human pathogen responsible for causing Visceral Leishmaniasis. Unavailability of proper treatment in the developing countries has served as a major threat to the people. The absence of vaccines has made treatment possibilities to rely solely over chemotherapy. Also, reduced drug efficacy due to emerging resistant strains magnifies the threat. Despite years of formulations for an effective drug therapy, complexity of the disease is also unfortunately increasing. Absence of potential drug targets has worsened the scenario. Therefore exploring new therapeutic approach is a priority for the scientific community to combat the disease. One of the most reliable ways to alter the adversities of the infection is finding new biological targets for designing potential drugs. An era of computational biology allows identifying targets, assisting experimental studies. It includes sorting the parasite’s metabolic pathways that pins out proteins essential for its survival. We have directed our study towards a computational methodology for determining targets against *L. donovani* from the “purine salvage” pathway. This is a mainstay pathway towards the maintenance of purine amounts in the parasitic pool of nutrients proving to be mandatory for its survival. This study represents an integration of metabolic pathway and Protein-Protein Interactions analysis. It consists of incorporating the available experimental data to the theoretical methods with a prospective to develop a kinetic model of Purine salvage pathway. Simulation data revealed the time course mechanism of the enzymes involved in the synthesis of the metabolites. Modeling of the metabolic pathway helped in marking of crucial enzymes. Additionally, the PPI analysis of the pathway assisted in building a static interaction network for the proteins. Topological analysis of the PPI network through centrality measures (MCC and Closeness) detected targets found common with Dynamic Modeling. Therefore our analysis reveals the enzymes ADSL (Adenylosuccinate lyase) and IMPDH (Inosine-5′-monophosphate dehydrogenase) to be important having a central role in the modeled network based on PPI and kinetic modeling techniques. Further the available three dimensional structure of the enzyme “ADSL” aided towards the search for potential inhibitors against the protein. Hence, the study presented the significance of integrating methods to identify key proteins which might be putative targets against the treatment of Visceral Leishmaniasis and their potential inhibitors.

## Introduction

One of the primary problems arising worldwide is the increasing risk of parasitic disease mostly infecting the people and animals in the underdeveloped countries (El Kouni, 2003). It includes infections from a wide source of microorganisms like fungi, bacteria, and protozoans etc. (Wang, 1984; Kokina et al., 2019). One such parasitic disease, Visceral leishmaniasis (VL or Kala Azar), caused by the protozoan species *Leishmania donovani* has served as a major threat to these countries, increasing the rate of fatality (Desjeux, 2004; Alvar et al., 2012). VL serves to be one of the most severe forms of leishmaniasis (Sundar, 2001) with the highest death rate (Cavalli and Bolognesi, 2009). This species can infect the internal organs threatening the human health (Sharma et al., 2017). Mostly affected are the poor people from the East African and the Indian subcontinent hence leading to a higher demand for the identification, treatment and control of the infection in the low and middle income countries (Chappuis et al., 2007).

Treatment of *Leishmania* infection relies on chemotherapy (Sundar and Chatterjee, 2006), however, failure towards the available chemotherapeutic agents and treatments still prevails. The first drugs for the treatment were made available around five decades ago. However, the formulation of a single drug is not sufficient to combat the species due to the differences in drug sensitivity among the *Leishmania* sp. (Croft and Coombs, 2003). The substantial side effects (Vijayakumar and Das, 2018) and difficulty in administration has also led to the evolution of drug resistant parasitic strains contributing to the increased rate of mortality (Sundar, 2001). Further, expensive treatment strategies acts as a hurdle towards an effective drug development (Bora, 1999; Croft et al., 2005). In a conclusive manner, a major challenge still exists in identifying effective cure and treatment for the parasite disease (Freitas-Junior et al., 2012) which requires an exploitation of current technologies for identifying novel chemotherapeutics (Davis et al., 2004). The mandate is to find novel drug-targets from the parasite’s proteome (Guerin et al., 2002). The identification of such targets from a pathogen’s biological pathway is reported to be an important feature in the drug discovery process (Chawla and Madhubala, 2010). This has helped in exploring ways for studying the protozoan’s metabolic pathways to sort out the ones unique to them (Martin et al., 2016). The information embedded in the microbe’s life cycle may pave the way for understanding the pathogenesis (Smith and Romesberg, 2007). It proves to be essential in controlling the microbial infections that are becoming resistant to the drugs available for their treatment causing fatal conditions.

One of the key factors in understanding the pathogen’s biological pathway requires knowledge of the underlying kinetics governing the enzymes and molecules involved in the pathway. This complex biological system can be represented into a network of interconnecting links signifying the reactions involved in the pathway (Meshram et al., 2019). *In silico* analysis of metabolic pathways through systems biology approach has been on the forefront for providing the means to understand the whole network through the availability of the experimental data. Hence, availability of experimental details paves a way for describing the pathway mathematically (Van Riel, 2006). System level analysis has been used as a tool for identifying targets against different species of *Leishmania* (Chavali et al., 2008; Mandlik et al., 2012; Sharma et al., 2017). However, providing a detailed mathematical model is still a challenge in systems biology (Steuer et al., 2006). Biological databases (Stein, 2003), provide a means to attain the knowledge of biological reactions involved in the pathways. Also with the growth of high throughput technologies (Baker and Brass, 1998), the size of these databases are increasing making the interpretation of data a major challenge in the scientific field (Guimera and Amaral, 2005).

Life cycle of *L. donovani* exists as flagellated extracellular promastigotes in the phlebotomine sandfly vector and as immotile intracellular amastigotes within cells of the infected mammalian host. The amastigote has been reported to be the cause behind the pathogen infections including Visceral Leishmaniasis (McConville et al., 2007). *L. donovani* lacks the machinery for the synthesis of purines competing with the hosts for salvaging the required purines (Boitz et al., 2012). Incapability of these organisms to synthesize the purines *de novo* has led to their dependence on the salvaging of the purines from their hosts rendering the “purine salvage” pathway essential for its survival (Boitz and Ullman, 2013; Ansari et al., 2016). Hence, the pathway has served has a backbone towards the maintenance of purine amounts in the parasitic pool of nutrients (Marr et al., 1978; Looker et al., 1983; De Koning et al., 2005). A lack of effective cure for the disease has made its importance in the scientific community opening up ways for delineation of this pathway (Boitz et al., 2012). The enzymes involved in this survival pathway opens up the exploration space for newer drug targets (Carter et al., 2008) for chemotherapeutic agents against parasitic diseases (Berg et al., 2010; Doleželová et al., 2018).

In the current work, we have carried out a twin approach – both dynamical and static, for identifying potential drug targets against the pathogen *L. donovani*. Unlike the traditional ways of identifying a target, we have carried out the *in silico* simulation of the “purine salvage” pathway for the protozoan which represents the dynamic method. The selected pathway has been represented in the form of a mathematical model defining the reactions catalyzed by the enzymes and the pathway components. Kinetic modeling of the pathway displayed the importance of the proteins Adenylosuccinate lyase (ADSL) and Inosine-5′-monophosphate dehydrogenase (IMPDH). The static method comprises of a Protein Protein Interaction (PPI) network for the proteins involved in the purine salvage pathway. PPI network analysis has been used as a tool for studying the proteomes of *Leishmania* species like *L. braziliensis*, *L. major* and *L. infantum* (Flórez et al., 2010; Rezende et al., 2012; Chávez-Fumagalli et al., 2018; Dos Santos Vasconcelos et al., 2018). The PPI networks are types of biological network representing a set of proteins and their interactions. It was carried out to analyze the importance of the sorted proteins from the dynamical model. A topological analysis of the PPI network showed that these proteins were also found to be ranked in the top central nodes (proteins) with higher connectivity. These nodes (hub proteins) have a higher number of connections that designates its physical associations with their other proteins responsible for performing the specialized functions. Hence a destruction of these nodes will lead to a loss of the connectivity and function which might be crucial for the parasite’s survival.

## Materials and Methods

A dual approach has been applied in our current work where a dynamic modeling and a static interaction network was generated. The dynamic modeling approach includes the selection of a pathway essential for the parasite survival, retrieving the enzymatic reactions and construction of a kinetic model reflecting the importance of certain enzymes which might be important targets in controlling *Leishmaniasis.* The static interaction network includes construction of a PPI and computing the hub nodes.

### Selection of Metabolic Pathway

A series of reactions are indulged within the parasite which effect the host (Lambris et al., 2008). These reactions are regulated by enzymes for the generation of the desired products. Some metabolic pathways of the pathogen are proven to be essential for the survival of the organism inside the host. Analysis of those reactions is crucial in listing out the enzymes which could be potential drug targets. One such pathway considered to be mandatory for the survival of *L. donovani* is the “purine salvage” pathway. The enzymes and reactions involved in the pathway are compiled with the help of the available resources.

### Biochemical Network Construction

#### Reactions of the Biological Pathway

The enzymes and the reactions involved in the salvaging of purines in *L. donovani* are obtained from the biological pathway resource “KEGG” (Kanehisa and Goto, 2000). The conversions of the metabolites were further cross checked with the current literature. For building of our model of interest, we have considered the reactions only occurring at the amastigote stage, i.e., the infection causing stage of the parasite (McConville et al., 2007). The metabolic pathway is then graphically represented through the pathway editor “Cell Designer” (Funahashi et al., 2003). It represents every reaction along with the substrates and the metabolites formed with a simpler view. The species transformation is represented through reactions (whether reversible or irreversible).

#### Kinetic Model

A kinetic rate law defines every reaction included in the model. Enzymatic mechanisms were studied to specify the rate equations for the reactions governed by the enzyme. For enzymes whose detailed mechanisms were not known, the rate equations were constrained to the basic Michaelis-Menten equation, with the basic knowledge that all enzymatic reactions follow the Michaelis-Menten equation. The kinetic model of our pathway is set up using the pathway simulator “COPASI” (Hoops et al., 2006). It constitutes the species defined in their biochemical terms. All the rate equations for the enzymes were generated through COPASI. The mathematical model of the metabolic pathway is defined in the form of Ordinary Differential equations (ODE). Defining a kinetic model implies retrieving available kinetic parameters through different sources. These parameters for the enzymes in our model is collected from curated enzyme database “Brenda” (Schomburg et al., 2004). Parameters not available in the database are collected through literature. Parameters unavailable for a few enzymes for *L. donovani* are taken from other reference organisms (Franco and Canela, 1984) assuming that the enzyme mechanisms remains conserved across species. Further integrating a system requires setting up the initial conditions. Considering this, we have set the initial concentrations for the different species in our model.

### Model Simulation and Analysis

The kinetic behavior of the model has been assessed through evaluating the species with respect to time.

#### Steady State Analysis

Initially the steady state analysis was carried out for our model using COPASI. It enables to analyze the state at which the concentrations of the species do not vary with respect to time. The behavior of the system at that state tends to continue to be present in the future. Methods that COPASI uses for the steady state calculations are the damped Newton method and the integration method both forward and backward.

#### Time Course Analysis

Followed by the steady state calculation we conducted the time course analysis using the “time course utility” of COPASI. The deterministic method (LSODA) is used for simulating the dynamic behavior and carried out for a period of 500 sec.

#### Sensitivity Analysis

Assessing the effects of the parameters over the system variables is another crucial point in kinetic modeling. This was carried out through a sensitivity analysis of the model using the sensitivity utility of COPASI.

### Protein-Protein Interaction Network

Enzymes sorted through our dynamic analysis were further checked for their importance in the pathway through a topological analysis of the Protein-Protein Interaction (PPI) Network. Compared to the kinetic models, the PPI represents the static interactions of a protein with their co-partners. These networks provide as an effective means towards understanding functional interactions, identification of important modules and prioritization of targets. Protein-Protein Interaction network are mathematically created networks where every protein is represented as a node and the interaction between two proteins as an edge. The functional protein interactions for *L. donovani* were retrieved from the available source of interaction database “STRING version 10.5” (Szklarczyk et al., 2014). The corresponding interactions of proteins involved in the purine salvage pathway were retrieved. Using this molecular interaction information, a Protein-Protein interaction (PPI) network has been constructed through the open source platform “Cytoscape” (Shannon et al., 2003) which represents the overall interaction of the selected proteins in the *L. donovani* sp. MCODE (Chin et al., 2014) clustering algorithm was applied to identify the clusters consisting of densely interconnected nodes. Proteins in a cluster tend to functionally link to each other. Topological analysis for the constructed PPI network was carried out to deduce the properties of the network. Central nodes in the network were analyzed through the Cytohubba (Chin et al., 2014) package of “Cytoscape.” These nodes show a higher number of connections with the other proteins in the network and hence maintain their associated functions. Deletion of the proteins behaving as the central nodes will lead to a loss of connectivity in the network. Hence, nodes having a higher tendency to alter the topology of the network are identified. They might prove to be important targets for inhibiting the growth and survivability of the pathogen.

### Inhibitor Search for the Selected Enzyme

The presence of the three dimensional structure of a protein assists towards the identification of possible inhibitors against the selected enzyme (Choudhary et al., 2019). Inhibitor search for our selected protein was carried out through pharmacophore mapping of the available ligands against the protein. In our study we highlighted two enzymes having a potential of being targets against *L. donovani*. However, the crystal structure of only the ADSL enzyme was available and hence we selected this enzyme for further study. A literature search was conducted to list out the possible inhibitors for the ADSL enzyme. These inhibitors were subjected to a pharmacophore generation through Pharmagist. The pharmacophore was then mapped against the ZINC database through the Zinc Pharmer to select out possible inhibitors for ADSL enzyme. A set of molecules were then selected from the database of molecules which were further considered for analysis. Molecular docking approach was applied to the selected molecules to observe the binding behavior of the ligands within the receptor molecule. Autodock version 4.2.6 was applied to carry out the molecular docking.

## Results and Discussion

### Selection of Metabolic Pathway

**FIGURE 1:**
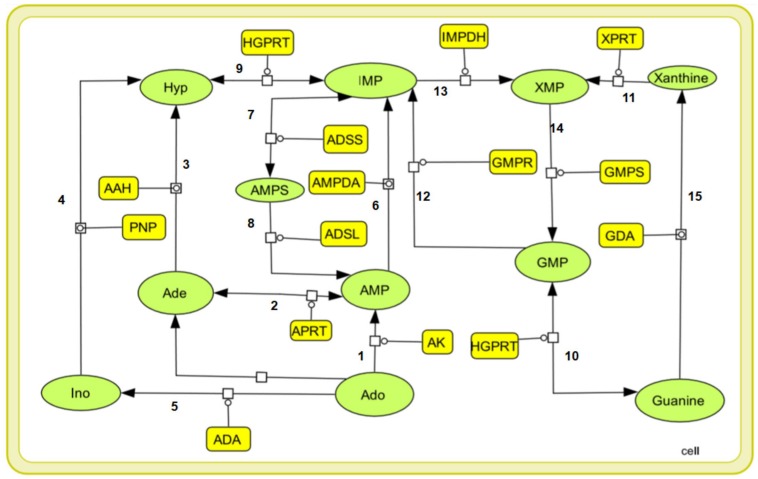
A graphical representation of the purine salvage pathway. The enzymes are shown in yellow rectangular boxes and the oval shapes represents the metabolites: Ade, adenine, Hyp, hypoxanthine, Ino, inosine, Ado, adenosine, AMP, IMP, XMP, GMP, Xanthine, Guanine, and AMPS.

Purine nucleotides are considered important for maintaining the vital processes of the cell. However, the *Leishmania* parasite being unable to synthesize the purine rings has developed a pathway for scavenging the required purines (Boitz et al., 2012). Literature sources showed various reactions in the incorporation of purines from the host metabolism. Summarizing all the possible transformations of the metabolites, a generalized form of the pathway is constructed including all the reaction products and the enzymes involved in salvaging of the nutrients. Also, the complexity of simulating kinetic models assists the fact that single pathways are more feasible to study. The main strategy while building our model was to reduce the system while still maintaining the overall characteristics of the biological system. Therefore in our current study we have considered a total of 15 reactions with 14 enzymes found to be involved in these reactions generating the desired metabolites. These enzymes are tabulated in Table 1 along with their reactions retrieved from BRENDA and literature. Several classes of enzymes like lyases, kinases, transferases etc. are found to be involved in the scavenging process which is shown by their EC numbers. The schematic representation of the pathway is created through Cell Designer. Both reversible and irreversible reactions were included in our model. The reactions and conversions playing an important role in the regulation of the metabolites are schematically arranged. These enzymes (Table 1) along with the reactions they catalyze are shown in Figure 1.

**TABLE 1:** Enzymes involved in the purine salvage pathway.

**Sl. No**	**Enzymes**	**EC No.**	**Reactions**
1	Adenosine kinase	2.7.1.20	Adenosine + ATP ->adenosine 5′ monophosphate + ADP
2	Adenine phosphoribosyltransferase	2.4.2.7	AMP + diphosphate = adenine + 5-phospho-alpha-D-ribose 1-diphosphate
3	Adenine aminohydrolase	3.5.4.2	Adenine + H_2_O → hypoxanthine + NH_3_
4	Purine nucleoside phosphorylase	3.2.2.1	Purine nucleoside + phosphate ⇌ purine + alpha-D-ribose 1-phosphate
5	Adenosine deaminase	3.5.4.4	Adenosine + H_2_O → inosine + NH_3_
6	AMP deaminase	3.5.4.6	AMP → IMP
7	Adenylosuccinate Synthase/Succino-AMP synthetase	6.3.4.4	GTP + IMP + L-aspartate = GDP + phosphate + adenylosuccinate
8	Adenylosuccinate lyase/Succino-AMP lyase ADSL	4.3.2.2	Succino-AMP = AMP + fumarate
9	Hypoxanthine-Guanine phosphoribosyltransferase	2.4.2.8	(1) IMP + Diphosphate = Hypoxanthine + 5-phospho-alpha-D-ribose 1-diphosphate (2) GMP + Diphosphate = Guanine + 5-phospho-alpha-D-ribose 1-diphosphate
10	Xanthine phosphoribosyltransferase	2.4.2.22	XMP + Diphosphate = 5-phospho-alpha-D-ribose 1-diphosphate + xanthine
11	GMP reductase	1.7.1.7	GMP + NADPH + (H+) → IMP + (NADP+) + NH_3_
12	IMPDH	1.1.1.205	IMP + (NAD+) + H_2_O → XMP + NADH + (H+)
13	GMP synthase	6.3.5.2	ATP + XMP + NH_3_ → AMP + diphosphate + GMP
14	Guanine deaminase	3.5.4.3	Guanine + H_2_O → xanthine + NH_3_

### Model Building

The kinetic model of the pathway was generated through the pathway simulation software “COPASI.” Pathway species (enzymes, metabolites) were incorporated to generate the biochemical model followed by fixing the defined rate laws against each enzyme. The rate of the enzymes was described by the Michaelis-Menten equations which were able to capture the necessary details of the reactions and hence displaying the dynamic behavior. These rate equations depend on the concentrations of the metabolites and parameters like binding constants, maximum velocity etc. The model was subsequently fed with the collected kinetic parameters setting up their initial concentrations. This yielded a set of ODE’s representing the enzyme kinetics of the system. Kinetic modeling solves these set of ODE’s for their dynamic behavior. Once the model has been assembled, it is simulated for observing the variations in the system. The publicly available biochemical network simulator “COPASI” was used to simulate the constructed model and also to perform the analysis.

### Model Simulation and Analysis

#### Steady State Analysis

A characteristic feature of metabolic network is to evolve into a steady state frequently. Computational calculation of steady state requires solving the model through solving the system of ODE’s numerically. Steady states of a system represent a certain physiological condition of the network. Changes in the physiological conditions results in transient states as the system transfers itself to a new steady state. The steady state of our kinetic model was calculated and it showed that the model acquired a steady state with our initial conditions and set of parameters. The steady state fluxes have been shown in Table 2.

**TABLE 2:** Steady state fluxes of Purine salvage model.

**Reactions**	**Enzymes**	**Flux (mol/s)**
1	Adenosine kinase	−1.00449*e*-15
2	Adenine phosphoribosyltransferase	4.77811*e*-15
3	Adenine aminohydrolase	3.22322*e*-15
4	Purine nucleoside phosphorylase	−4.20221*e*-14
5	Adenosine deaminase	1.78269*e*-15
6	AMP deaminase	3.90643*e*-18
7	Adenylosuccinate Synthase/Succino-AMP synthetase	6.24077*e*-15
8	Adenylosuccinate lyase/Succino-AMP lyase ADSL	6.0136*e*-15
9	Hypoxanthine-Guanine	−6.8775*e*-15
10	phosphoribosyltransferase	−1.68639*e*-15
11	Xanthine phosphoribosyltransferase	3.02017*e*-12
12	GMP reductase	−1.80154*e*-15
13	IMPDH	4.72791*e*-16
14	GMP synthase	7.62529*e*-20
15	Guanine deaminase	−2.32796*e*-15

#### Time Course Analysis

**FIGURE 2:**
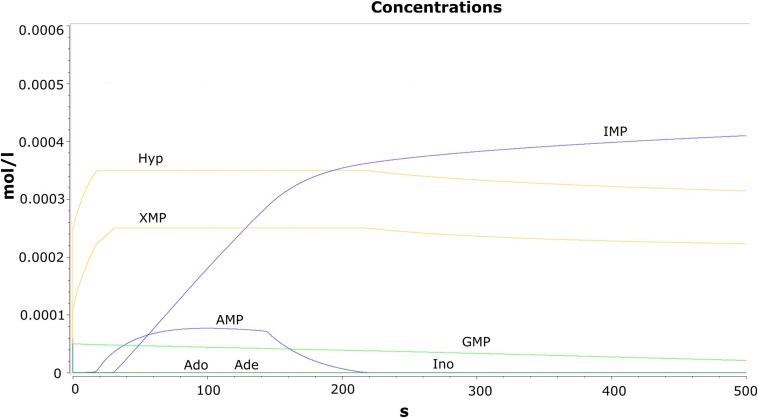
Results of the time course analysis for the metabolites.

Time evolution of the model was carried out through the “time course analysis” utility of COPASI. The main objective of performing this analysis is to observe the dynamics of the model. A graph shows the behavior of the model with respect to time (Figure 2). The concentration vs. time plot is generally used to infer the attainment of a steady state. A plateau has been observed in the trajectories of the concentrations against time plot where no further changes in the variables are observed. This suggests that the model has reached the steady state characterized by the constant species concentrations. The production and the consumption of the metabolites occur at the same rate.

#### Sensitivity Analysis

**FIGURE 3:**
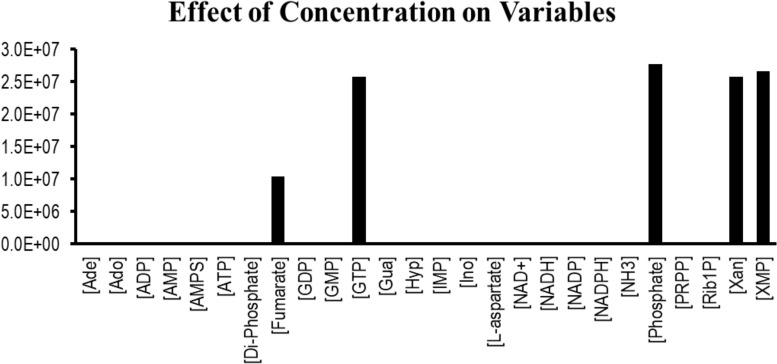
Effect of the species concentration over all the variables in the model.

**FIGURE 4:**
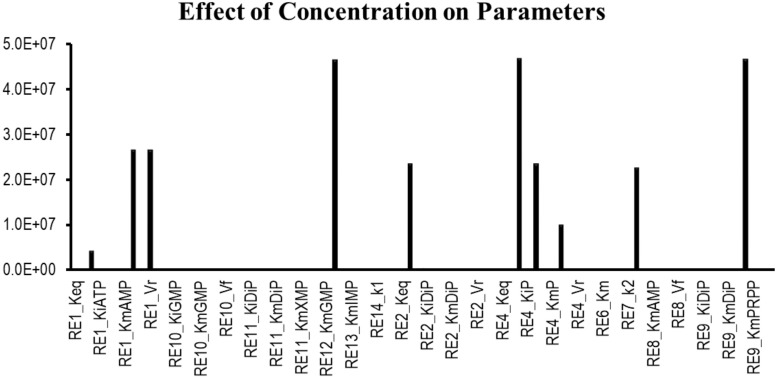
Sensitivity of the parameters involved in the model.

Sensitivity analysis is essentially performed to observe the consequences of the parameters over the model variables like concentration of the species. Two plots were generated showing the effects of the concentration of the species over the variables and the parameters. Figure 3 displays that the reactions catalyzed by the enzymes IMPDH, ADSL, ADSS, XPRT, GDA, and GMPS were sensitive to the initial concentrations of the marked variables. Whereas Figure 4 shows that the marked parameters involved in the reactions 1, 2, 4, 8, 9, and 12 were mostly sensitive to the model. These reactions were catalyzed by the enzymes AK, APRT, PNP, ADSL, HGPRT, and the IMPDH, respectively. It is observed from both the cases that the enzymes IMPDH and ADSL are the most sensitive to the effect of concentration. Therefore, the study indicates that these specific reactions probable to be sensitive in the metabolic chain could be further analyzed through future experiments to give valuable insights into the dynamics of the system. Moreover the experimental studies could be directed towards the therapeutic importance of these reactions. Thus, the behavior observed here provides generous amount of dynamics of the “Purine salvage” pathway reflecting the importance of these reactions.

### Protein-Protein Interaction Network

To further analyze the importance of the two proteins, IMPDH and ADSL, we carried out a static analysis along with the dynamics study. The available protein-protein interactions for *L. donovani* from STRING consists a total of 3707 proteins and a total of 437053 interactions. The constructed PPI network of the purine salvage pathway showed the participation of 1581 proteins (nodes) and 5735 unique interactions (list of proteins attached at the end of Supplementary Material). The interactions showed proteins to be involved in other functions other than the purine salvage. Clustering of the network through MCODE resulted into two clusters as tabulated in Table 3. Comparison of both the clusters reveals that lesser number of proteins were involved with a higher number of interactions resulting into a dense network. Proteins in a dense network often forms functional modules that contributes to cellular processes (Rasti and Vogiatzis, 2019).

**TABLE 3:** MCODE clustering result.

**Clusters**	**No. of nodes**	**No. of nodes**	**Score**
Cluster 1	27	195	15
Cluster 2	67	143	4.333

Therefore knocking out of these proteins should result into a much lesser number of interactions which will lead to an overall loss of a number of functions of the proteins. These interactions may also prove to be fatal. On the other hand, in the second cluster higher number of proteins were involved having lesser number of interactions compared to cluster 1. The proteins which were found to be sensitive in the kinetic modeling approach were analyzed for their presence in the cluster. The enzymes ADSL and IMPDH were found to be having interactions with other proteins in the first cluster. Along with these two proteins, two other proteins were also found to be present in the interaction pattern. These proteins along with their STRING ids have been tabulated in Supplementary Table S1. Cluster 1 of the MCODE clustering method has been shown in Supplementary Figure S1 with the tabulated enzymes being highlighted in the network. The other protein APRT (Adenine phosphoribosyltransferase) with a STRING id of XP_003861601.1 was found to be present in cluster 2 (Supplementary Figure S2). Rest of the proteins were not involved in formation of any interactions in these two clusters.

Network analysis enhances our understanding of the interactions of a node with other nodes in the network. As the biological network is heterogeneous, different topological parameters were used to identify the essential proteins. The MCC (Maximum Clique centrality) method of Cytohubba is reported to be a better method in identifying central nodes (Chin et al., 2014). It shows that the proteins ADSL and IMPDH were captured to be the central nodes. Another method to rank the proteins applied was global centrality based method “closeness.” It showed that the two proteins IMPDH and ADSL were ranked to be essential.

Literature studies support the fact the enzyme IMPDH is a promising target for drug discovery in antibacterial, anticancer and antiviral treatments (Shu and Nair, 2008) and organism like *Pneumocystis carinii* (O’Gara et al., 1997). Further importance of this enzyme has also been demonstrated in the organism *Leishmania amazonensis* (Pitaluga et al., 2015). ADSL is known as an important target in organisms like *Plasmodium falciparum* (malaria) (Bulusu et al., 2009), *Cryptococcus neoformans* (Chitty et al., 2017), *Staphylococcus aureus* (Fyfe et al., 2010) and *Schistosoma mansoni* (schistosomiasis) (Romanello et al., 2017). Experimental groups have also reported the fact that a decrease growth rate occurs for the phenotypes when the ADSL gene is knocked out (Boitz et al., 2013). Therefore, studies could be further conducted upon targeting these proteins of interest which might lead to a loss of *Leishmania* infections (Galina et al., 2017). *In silico* mutational analysis of the ADSL protein suggested a few mutations that bought conformational changes to the catalytic site of the protein (Bora and Nath Jha, 2019).

### Inhibitor Search for the Selected Enzyme

Availability of three dimensional structures for the proteins gives an insight into the molecular information of the proteins (Das et al., 2018). Exploring the protein structural repository “PDB”, it was found that the crystal structure for only ADSL (PDB id: 4MX2) was available. This led to the exploration of the protein through other computational techniques highlighting it as a probable target. We carried out an analysis to identify potent inhibitors against this target molecule having an available 3D structure.

**FIGURE 5:**
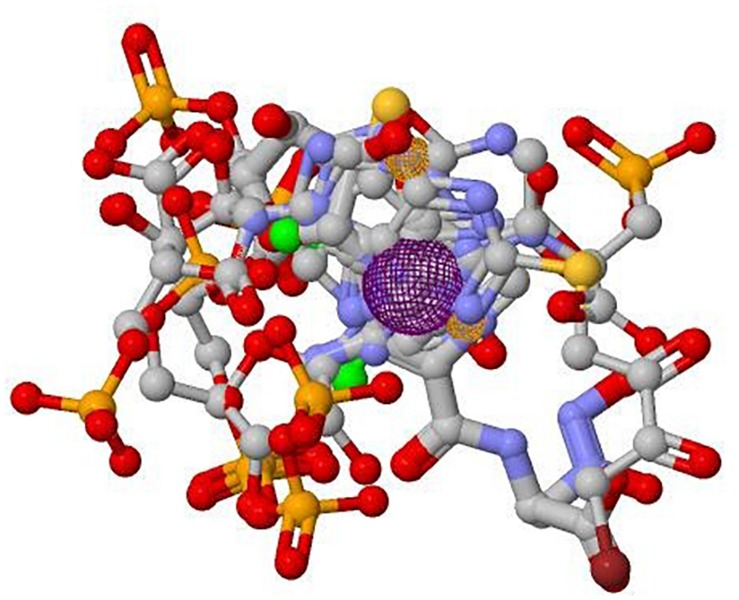
Pharmacophore of the known inhibitors against ADSL.

A total of 14 molecules reported to be possible inhibitors of Adenylosuccinate Lyase has been listed out (shown in Supplementary Table S2). Pharmacophore generation of the 14 molecules was carried out to find out the potential regions of the ligands contributing towards the catalytic activity. It was found out that 11 molecules were best aligned in generating the desired pharmacophore. Three chemical features were found out to be involved in maintaining the activity of the ligand molecules which includes two hydrogen bond acceptor regions and one aromatic region. The generated pharmacophore has been shown in Figure 5. It was then used as a query to search potential ligands. The reason behind the use of searching the database through a pharmacophore is to identify hits having similar chemical features to that of the query. We mapped the pharmacophore against the Zinc database. Twenty inhibitors with a root mean square deviation (rmsd) of zero were selected out as our dataset for further analysis. The list of molecules is tabulated in Supplementary Table S3. The molecules were found to be satisfying Lipinski’s rule which designates the druglike property of the molecules. To find out the optimal binding pose of these ligands to the enzyme, the ligand molecules were docked to the active site of the ADSL enzyme. The results of the Docking have been shown in Supplementary Table S4.

**FIGURE 6:**
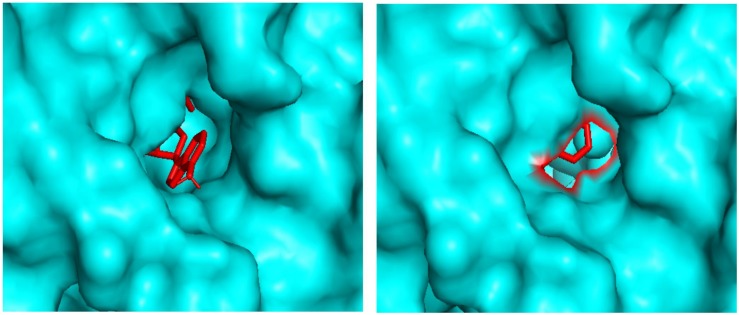
Docking pictures of Molecule 7 and Molecule 13 within the active site of ADSL.

Molecular docking analysis revealed the binding patterns of the ligand molecules within the ADSL protein. Docking results showed that the binding energy of all the molecules were quite stable but out of 20 molecules, 18 molecules were found to be forming hydrogen bonds with the receptor. The strength of binding usually depends of the interaction between the ligand atoms and the receptor atoms. Therefore the presence of hydrogen bonds aids toward the stability of the ligand-receptor complexes. Also out of the 18 molecules, 12 molecules were observed to be forming hydrogen bonds with the active site residues. These residues are reported to be involved in the catalysis process and hence the binding of these ligands to the residues showcases their importance in inhibiting the enzyme. Also molecule 7 and molecule 13 has been observed to be interacting with the maximum number of active residues. The orientation of the molecules inside the cavity of the protein has been shown in Figure 6. Results showed that the molecules were able to bind deep within the activity that assists them in their catalytic properties. Therefore the effective binding of the molecules to the protein display their therapeutic importance as drug molecules.

## Conclusion

The *in silico* study reflected that a systems level analysis for a protozoan parasite “*L. donovani*” is possible resulting into the identification of drug targets. In our work, a biochemical network of a metabolic pathway “Purine salvage” for the organism *L. donovani* has been constructed. The dynamic behavior of the model is analyzed through a mathematical representation of the reactions showcasing the biological events. The dynamic simulation allowed us to define the biological pathway of interest with respect to time. Further the model stands as a benchmark for incorporation of complex enzymatic mechanisms through the availability of experimental data. Reactions showing higher sensitivity to the model have been sorted out with the listing of the enzymes regulating those reactions. These enzymes and their reactions could be further experimentally tested out for their importance in therapeutics. Also to analyze the essentiality of these proteins, a static interaction network (the PPI network) for the proteins involved in purine salvage has been constructed. Clustering analysis resulted into a much higher dense network consisting of the proteins ADSL and IMPDH. It was observed through topological analysis that the mentioned proteins were ranked among the top 30% of the hub proteins. These proteins might serve as targets to further explore the pathway mechanism shedding light on the control of infections caused by the pathogens. Availability of the three dimensional structures for ADSL marks its possibility for other computational analysis like Virtual screening of inhibitors. Search for potential inhibitors for the ADSL protein was carried out through the identification of specific chemical features i.e., the pharmacophore. Mapping of the pharmacophore to available ligands lead to the selection of molecules having similar features and within minimum variation from the query molecules. To observe the binding mode of the ligand molecules to that of the receptor, molecular docking analysis was applied. Results of the docking analysis showed that two molecules were able to effectively bind to the active site residues of the protein. Hence it displayed their therapeutic importance as inhibitors having features similar to the known inhibitors against ADSL.

## Data Availability Statement

The datasets generated for this study are available on request to the corresponding author.

## Author Contributions

NB performed the *in silico* studies, analyzed the data, and wrote the manuscript. AJ supervised this study.

## Conflict of Interest

The authors declare that the research was conducted in the absence of any commercial or financial relationships that could be construed as a potential conflict of interest.
