# Supplementary material for: In silico Metabolic Pathway Analysis Identifying Target Against Leishmaniasis – A Kinetic Modeling Approach
Source: Front Genet. 2020 Mar 6;11:179. doi: 10.3389/fgene.2020.00179 (PMC7068213; doi:10.3389/fgene.2020.00179)
Supplement: Supplementary file 1 [file Table_1.DOCX]

**Supplementary File**

**"*In-silico* metabolic pathway**

**Analysis identifying target against Leishmaniasis – A Kinetic modeling approach"**

Nikita Bora^1^, Anupam Nath Jha^1*^

^1^ Computational Biophysics Laboratory, Department of Molecular Biology and Biotechnology,

Tezpur University, Tezpur – 784028, Napaam, Sonitpur, Assam, India.

*To whom correspondence should be addressed:

**Corresponding Author:** Anupam Nath Jha

Tel.: +91 3712-275416

Fax: +91 3712-267005

Email: [anjha@tezu.ernet.in](mailto:anjha@tezu.ernet.in)

**Supplementary Tables**

**Table 1: Proteins from dynamic analysis found to be involved in cluster 1**

| **S. No** | **Proteins** | **Name** | **STRING IDs** |
| --- | --- | --- | --- |
| 1 | ADSL | Adenylosuccinate lyase | XP_003858107.1 |
| 2 | IMPDH | Inosine-5’-monophosphate dehydrogenase | XP_003860332.1 |
| 3 | HGPRT | Hypoxanthine-guanine phosphoribosyltransferase | XP_003860615.1 |
| 4 | AK | Adenosine kinase | XP_003862807.1 |

**Table 2: List of inhibitors against ADSL enzyme**

| **Molecules** | **Name** |
| --- | --- |
| 1 | 6-Thioinosine 5'-Phosphate |
| 2 | AICAriboside |
| 3 | L-Alanosyl-5-aminoimidazole-4-carboxylic acid ribonucleotide (alanosyl-AICOR) |
| 4 | 5-Amino-4-imidazolecarboxamide ribotide (AICAR) |
| 5 | AMP |
| 6 | ATP |
| 7 | trans-4-hydroxy-2-nonenal (HNE) |
| 8 | Nonanal |
| 9 | N-ethyl maleimide |
| 10 | iodoacetamide |
| 11 | 2-[(4-bromo-2,3-dioxobutyl)thio]adenosine 5‘-monophosphate (2-BDB-TAMP) |
| 12 | 6-mercaptopurine nucleotide (THIOINOSINATE) |
| 13 | (7V6-(dl- 1 -carboxy-2-phosphonoethyl)- adenosine S'-monophosphate (adenylophosphonopropionate) |
| 14 | succinoadenine |

**Table 3: Dataset of molecules**

| **S. No** | **ZINC ID** | **Names** |
| --- | --- | --- |
| 1 | ZINC95363868 | [(3R,4R)-4-[(4-ethylpiperazin-1-yl)methyl]-1-(9H-purin-6-yl)pyrrolidin-3-yl]methanol |
| 2 | ZINC71759992 | (2S)-2-(2,2-diphenylethyl)-4-(9H-purin-6-yl)morpholine |
| 3 | ZINC76935763 | 3-methyl-3-phenyl-N-[[(2R)-4-(9H-purin-6-yl)morpholin-2-yl]methyl]butanamide |
| 4 | ZINC89761639 | 3-methyl-3-phenyl-N-[[(2R)-4-(9H-purin-6-yl)morpholin-2-yl]methyl]butanamide |
| 5 | ZINC81190927 | N-[(3S,5S)-5-methyl-1-phenyl-pyrrolidin-3-yl]-2-[methyl(7H-purin-6-yl)amino]acetamide |
| 6 | ZINC03995399 | BRD-A35338386-001-04-6 |
| 7 | ZINC05034293 | methyl |
| 8 | ZINC09418725 | azepan-1-yl-[1-(9H-purin-6-yl)-3-piperidyl]-methanone |
| 9 | ZINC91332525 | 9-[3-[(1S,2R)-2-methylcyclohexoxy]propyl]purin-6-amine |
| 10 | ZINC20744556 | (4R)-4-(2,4-difluorophenyl)-3-methyl-1-(9H-purin-6-yl)-5,7-dihydro-4H-pyrazolo[4,5-e]pyridin-6-one |
| 11 | ZINC31810217 | (4R)-4-(2,6-difluorophenyl)-3-methyl-1-(9H-purin-6-yl)-5,7-dihydro-4H-pyrazolo[3,4-b]pyridin-6-one |
| 12 | ZINC31811749 | (4R)-4-(2-isopropoxyphenyl)-3-methyl-1-(9H-purin-6-yl)-5,7-dihydro-4H-pyrazolo[3,4-b]pyridin-6-one |
| 13 | ZINC20739786 | (4R)-4-(2,3-dimethoxyphenyl)-3-methyl-1-(9H-purin-6-yl)-5,7-dihydro-4H-pyrazolo[4,5-e]pyridin-6-one |
| 14 | ZINC31810988 | (4S)-4-(3-chlorophenyl)-3-methyl-1-(9H-purin-6-yl)-5,7-dihydro-4H-pyrazolo[3,4-b]pyridin-6-one |
| 15 | ZINC40512421 | N-[(2R)-2-morpholino-2-phenyl-ethyl]-7H-purin-6-amine |
| 16 | ZINC95408255 | 9-[3-[4-(2-methyl-1H-indol-3-yl)-1-piperidyl]propyl]purin-6-amine |
| 17 | ZINC20736205 | (4S)-4-(3-hydroxy-4-methoxy-phenyl)-3-methyl-1-(9H-purin-6-yl)-5,7-dihydro-4H-pyrazolo[4,5-e]pyridin |
| 18 | ZINC31807861 | (4S)-4-(3,4-difluorophenyl)-3-methyl-1-(9H-purin-6-yl)-5,7-dihydro-4H-pyrazolo[3,4-b]pyridin-6-one |
| 19 | ZINC31811630 | (4S)-3-methyl-1-(9H-purin-6-yl)-4-[3-(trifluoromethyl)phenyl]-5,7-dihydro-4H-pyrazolo[3,4-b]pyridin- |
| 20 | ZINC20736212 | (4R)-4-(2-allyloxyphenyl)-3-methyl-1-(9H-purin-6-yl)-5,7-dihydro-4H-pyrazolo[4,5-e]pyridin-6-one |

**Table 4: Molecular Docking results**

| Molecules | Binding Energy | No. of Hydrogen Bonds | Residues involved in Hydrogen Bonding |
| --- | --- | --- | --- |
| 1 | -3.57 | 5 | ChainC: Arg 388, Arg 43, Asp 44, Asp 332  ChainD: **Asp 119** |
| 2 | -5.71 | 3 | Chain C: Arg 43, Arg388, Asp 44 |
| 3 | -2.89 | 3 | Chain C: Asn 329, Val 319  Chain D: **Asn 117** |
| 4 | -3.64 | 2 | Chain C: Asp 44, Arg 43 |
| 5 | -2.66 | 2 | Chain D: Glu113, **Asn 117** |
| 6 | -1.71 | 3 | Chain C: Glu 318, Asp 332  Chain D: **Asn 117** |
| 7 | -3.05 | 3 | Chain C: Asn 335  Chain D: **Ser 366**, **Asp 119** |
| 8 | -4.71 | 3 | Chain C: Arg 43, Asp 44, Arg 388 |
| 9 | -2.93 | 2 | Chain C: Asn 329  Chain D: **Asn 117** |
| 10 | -3.07 | - | - |
| 11 | -3.45 | - | - |
| 12 | -3.38 | 1 | Chain C: **Arg 40** |
| 13 | -4.57 | 3 | Chain C: Asn 329,  Chain D: **Asn 117**, **Ser 366** |
| 14 | -4.43 | 1 | Chain D: **Asn 117** |
| 15 | -4.06 | 2 | Chain C: Asp 44  Chain D: **Ser 366** |
| 16 | -3.06 | 1 | Chain D: Glu 113 |
| 17 | -3.07 | 4 | Chain C: Asp 44, Arg 43 (2)  Chain D: Glu 113 |
| 18 | -3.65 | 1 | Chain C : **Arg 40** |
| 19 | -2.67 | 1 | Chain C: Arg 315 |
| 20 | -4.45 | 3 | Chain C: **Arg 40**, Arg 43, Asp 44 |

*Residues in bold are the active site residues

**Supplementary Figures**


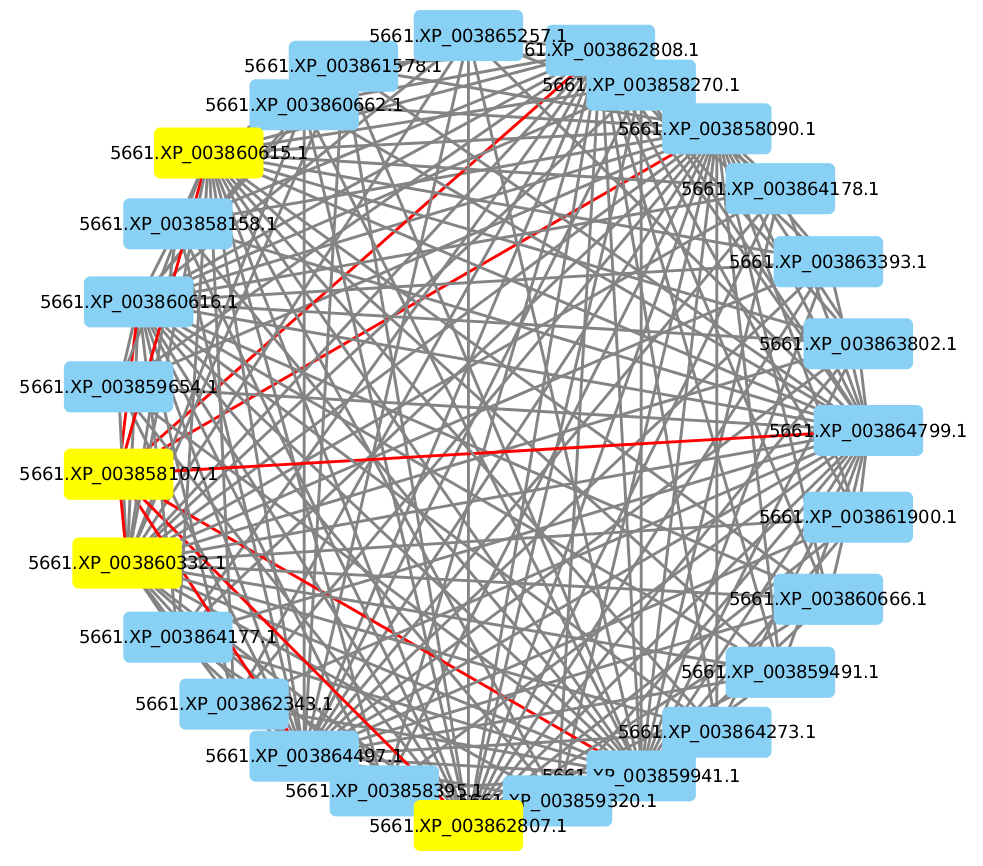


**Supplementary Figure 1:** Cluster1 of Mcode Clustering method. Yellow highlights the proteins that are found to be sensitive in the dynamics method. Red represents the associated interactions of the highlighted proteins.


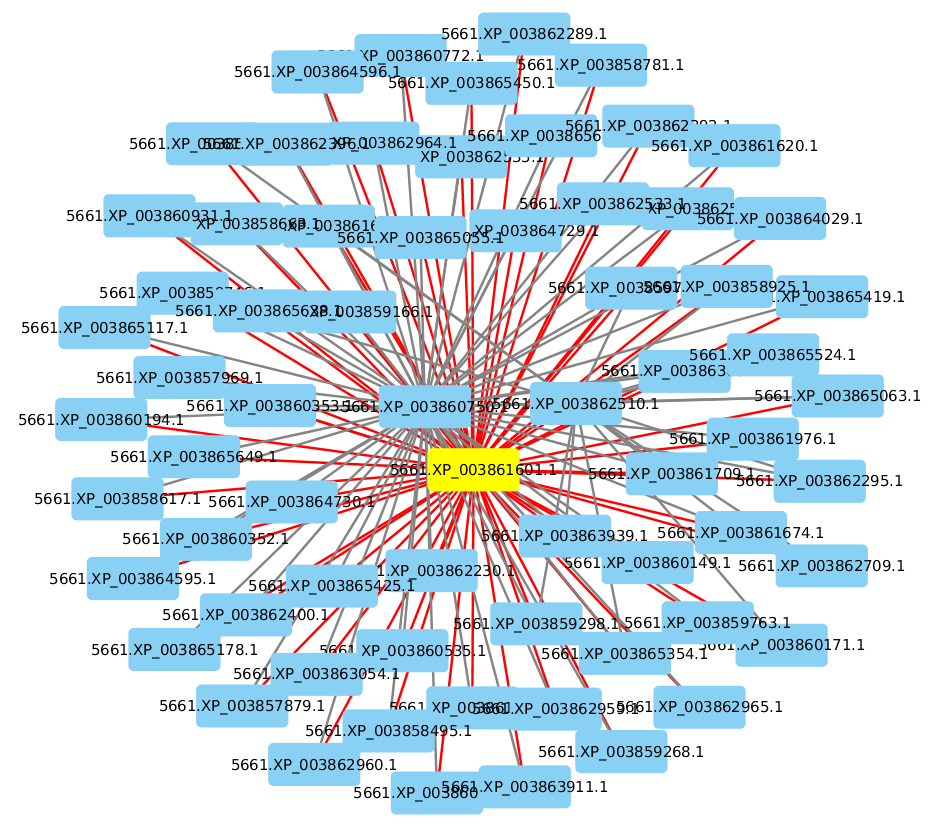


**Supplementary Figure 2**: Cluster 2 of Mcode Clustering method. Yellow highlights the proteins that are found to be sensitive in the dynamics method. Red represents the associated interactions of the highlighted proteins.

**List of Proteins (STRING Id’s) involved in Purine salvage PPI network**

| 5661.XP_003857831.1 | 5661.XP_003858065.1 | 5661.XP_003858288.1 | 5661.XP_003858556.1 | 5661.XP_003858911.1 |  |  |
| --- | --- | --- | --- | --- | --- | --- |
| 5661.XP_003857835.1 | 5661.XP_003858090.1 | 5661.XP_003858311.1 | 5661.XP_003858557.1 | 5661.XP_003858912.1 |  |  |
| 5661.XP_003857840.1 | 5661.XP_003858091.1 | 5661.XP_003858325.1 | 5661.XP_003858563.1 | 5661.XP_003858913.1 |  |  |
| 5661.XP_003857841.1 | 5661.XP_003858093.1 | 5661.XP_003858334.1 | 5661.XP_003858565.1 | 5661.XP_003858914.1 |  |  |
| 5661.XP_003857844.1 | 5661.XP_003858095.1 | 5661.XP_003858337.1 | 5661.XP_003858566.1 | 5661.XP_003858925.1 |  |  |
| 5661.XP_003857857.1 | 5661.XP_003858096.1 | 5661.XP_003858343.1 | 5661.XP_003858571.1 | 5661.XP_003858930.1 |  |  |
| 5661.XP_003857860.1 | 5661.XP_003858107.1 | 5661.XP_003858345.1 | 5661.XP_003858573.1 | 5661.XP_003858932.1 |  |  |
| 5661.XP_003857872.1 | 5661.XP_003858109.1 | 5661.XP_003858348.1 | 5661.XP_003858588.1 | 5661.XP_003858937.1 |  |  |
| 5661.XP_003857879.1 | 5661.XP_003858113.1 | 5661.XP_003858354.1 | 5661.XP_003858589.1 | 5661.XP_003858943.1 |  |  |
| 5661.XP_003857880.1 | 5661.XP_003858119.1 | 5661.XP_003858365.1 | 5661.XP_003858594.1 | 5661.XP_003858950.1 |  |  |
| 5661.XP_003857886.1 | 5661.XP_003858137.1 | 5661.XP_003858366.1 | 5661.XP_003858595.1 | 5661.XP_003858982.1 |  |  |
| 5661.XP_003857892.1 | 5661.XP_003858138.1 | 5661.XP_003858369.1 | 5661.XP_003858611.1 | 5661.XP_003858988.1 |  |  |
| 5661.XP_003857905.1 | 5661.XP_003858139.1 | 5661.XP_003858382.1 | 5661.XP_003858617.1 | 5661.XP_003858989.1 |  |  |
| 5661.XP_003857922.1 | 5661.XP_003858140.1 | 5661.XP_003858386.1 | 5661.XP_003858647.1 | 5661.XP_003858997.1 |  |  |
| 5661.XP_003857925.1 | 5661.XP_003858143.1 | 5661.XP_003858395.1 | 5661.XP_003858658.1 | 5661.XP_003858999.1 |  |  |
| 5661.XP_003857929.1 | 5661.XP_003858156.1 | 5661.XP_003858397.1 | 5661.XP_003858660.1 | 5661.XP_003859001.1 |  |  |
| 5661.XP_003857931.1 | 5661.XP_003858157.1 | 5661.XP_003858402.1 | 5661.XP_003858661.1 | 5661.XP_003859002.1 |  |  |
| 5661.XP_003857949.1 | 5661.XP_003858158.1 | 5661.XP_003858404.1 | 5661.XP_003858663.1 | 5661.XP_003859003.1 |  |  |
| 5661.XP_003857955.1 | 5661.XP_003858163.1 | 5661.XP_003858366.1 | 5661.XP_003858679.1 | 5661.XP_003859005.1 |  |  |
| 5661.XP_003857958.1 | 5661.XP_003858174.1 | 5661.XP_003858369.1 | 5661.XP_003858681.1 | 5661.XP_003859009.1 |  |  |
| 5661.XP_003857963.1 | 5661.XP_003858179.1 | 5661.XP_003858382.1 | 5661.XP_003858686.1 | 5661.XP_003859010.1 |  |  |
| 5661.XP_003857969.1 | 5661.XP_003858183.1 | 5661.XP_003858386.1 | 5661.XP_003858688.1 | 5661.XP_003859012.1 |  |  |
| 5661.XP_003857970.1 | 5661.XP_003858192.1 | 5661.XP_003858395.1 | 5661.XP_003858706.1 | 5661.XP_003859018.1 |  |  |
| 5661.XP_003857971.1 | 5661.XP_003858201.1 | 5661.XP_003858397.1 | 5661.XP_003858712.1 | 5661.XP_003859027.1 |  |  |
| 5661.XP_003857973.1 | 5661.XP_003858205.1 | 5661.XP_003858402.1 | 5661.XP_003858718.1 | 5661.XP_003859028.1 |  |  |
| 5661.XP_003857981.1 | 5661.XP_003858207.1 | 5661.XP_003858404.1 | 5661.XP_003858720.1 | 5661.XP_003859030.1 |  |  |
| 5661.XP_003857985.1 | 5661.XP_003858222.1 | 5661.XP_003858449.1 | 5661.XP_003858726.1 | 5661.XP_003859031.1 |  |  |
| 5661.XP_003857990.1 | 5661.XP_003858223.1 | 5661.XP_003858455.1 | 5661.XP_003858732.1 | 5661.XP_003859034.1 |  |  |
| 5661.XP_003858011.1 | 5661.XP_003858227.1 | 5661.XP_003858456.1 | 5661.XP_003858744.1 | 5661.XP_003859035.1 |  |  |
| 5661.XP_003858017.1 | 5661.XP_003858228.1 | 5661.XP_003858457.1 | 5661.XP_003858767.1 | 5661.XP_003859036.1 |  |  |
| 5661.XP_003858020.1 | 5661.XP_003858233.1 | 5661.XP_003858463.1 | 5661.XP_003858768.1 | 5661.XP_003859039.1 |  |  |
| 5661.XP_003858022.1 | 5661.XP_003858238.1 | 5661.XP_003858466.1 | 5661.XP_003858769.1 | 5661.XP_003859040.1 |  |  |
| 5661.XP_003858023.1 | 5661.XP_003858239.1 | 5661.XP_003858467.1 | 5661.XP_003858770.1 | 5661.XP_003859046.1 |  |  |
| 5661.XP_003858031.1 | 5661.XP_003858242.1 | 5661.XP_003858470.1 | 5661.XP_003858774.1 | 5661.XP_003859066.1 |  |  |
| 5661.XP_003858032.1 | 5661.XP_003858248.1 | 5661.XP_003858473.1 | 5661.XP_003858779.1 | 5661.XP_003859067.1 |  |  |
| 5661.XP_003858044.1 | 5661.XP_003858251.1 | 5661.XP_003858474.1 | 5661.XP_003858781.1 | 5661.XP_003859072.1 |  |  |
| 5661.XP_003858055.1 | 5661.XP_003858253.1 | 5661.XP_003858476.1 | 5661.XP_003858782.1 | 5661.XP_003859073.1 |  |  |
| 5661.XP_003858056.1 | 5661.XP_003858255.1 | 5661.XP_003858477.1 | 5661.XP_003858792.1 | 5661.XP_003859076.1 |  |  |
| 5661.XP_003858063.1 | 5661.XP_003858262.1 | 5661.XP_003858480.1 | 5661.XP_003858806.1 | 5661.XP_003859091.1 |  |  |
| 5661.XP_003858064.1 | 5661.XP_003858270.1 | 5661.XP_003858482.1 | 5661.XP_003858808.1 | 5661.XP_003859093.1 |  |  |
| 5661.XP_003858415.1 | 5661.XP_003858438.1 | 5661.XP_003858483.1 | 5661.XP_003858830.1 | 5661.XP_003859096.1 |  |  |
| 5661.XP_003858417.1 | 5661.XP_003858443.1 | 5661.XP_003858484.1 | 5661.XP_003858831.1 | 5661.XP_003859098.1 |  |  |
| 5661.XP_003858429.1 | 5661.XP_003858446.1 | 5661.XP_003858495.1 | 5661.XP_003858832.1 | 5661.XP_003859099.1 |  |  |
| 5661.XP_003858870.1 | 5661.XP_003865842.1 | 5661.XP_003858845.1 | 5661.XP_003859100.1 | 5661.XP_003859134.1 | |  |
| 5661.XP_003858892.1 | 5661.XP_003865838.1 | 5661.XP_003858847.1 | 5661.XP_003859101.1 | 5661.XP_003859137.1 | |  |
| 5661.XP_003858897.1 | 5661.XP_003865834.1 | 5661.XP_003858852.1 | 5661.XP_003859111.1 | 5661.XP_003859138.1 | |  |
| 5661.XP_003858898.1 | 5661.XP_003865832.1 | 5661.XP_003858855.1 | 5661.XP_003859114.1 | 5661.XP_003859166.1 | |  |
| 5661.XP_003858901.1 | 5661.XP_003858860.1 | 5661.XP_003859116.1 | 5661.XP_003859140.1 | 5661.XP_003859175.1 | | |
| 5661.XP_003858902.1 | 5661.XP_003858868.1 | 5661.XP_003859133.1 | 5661.XP_003859151.1 | 5661.XP_003859178.1 | | |

| 5661.XP_003859192.1 | 5661.XP_003859371.1 | 5661.XP_003859614.1 | 5661.XP_003859745.1 | 5661.XP_003860003.1 |
| --- | --- | --- | --- | --- |
| 5661.XP_003859193.1 | 5661.XP_003859373.1 | 5661.XP_003859618.1 | 5661.XP_003859746.1 | 5661.XP_003860006.1 |
| 5661.XP_003859198.1 | 5661.XP_003859374.1 | 5661.XP_003859619.1 | 5661.XP_003859751.1 | 5661.XP_003860008.1 |
| 5661.XP_003859202.1 | 5661.XP_003859385.1 | 5661.XP_003859626.1 | 5661.XP_003859755.1 | 5661.XP_003860016.1 |
| 5661.XP_003859204.1 | 5661.XP_003859396.1 | 5661.XP_003859628.1 | 5661.XP_003859756.1 | 5661.XP_003860018.1 |
| 5661.XP_003859211.1 | 5661.XP_003859401.1 | 5661.XP_003859630.1 | 5661.XP_003859760.1 | 5661.XP_003860022.1 |
| 5661.XP_003859217.1 | 5661.XP_003859404.1 | 5661.XP_003859634.1 | 5661.XP_003859763.1 | 5661.XP_003860029.1 |
| 5661.XP_003859220.1 | 5661.XP_003859405.1 | 5661.XP_003859636.1 | 5661.XP_003859774.1 | 5661.XP_003860037.1 |
| 5661.XP_003859228.1 | 5661.XP_003859407.1 | 5661.XP_003859640.1 | 5661.XP_003859775.1 | 5661.XP_003860041.1 |
| 5661.XP_003859238.1 | 5661.XP_003859414.1 | 5661.XP_003859644.1 | 5661.XP_003859783.1 | 5661.XP_003860043.1 |
| 5661.XP_003859251.1 | 5661.XP_003859417.1 | 5661.XP_003859645.1 | 5661.XP_003859786.1 | 5661.XP_003860047.1 |
| 5661.XP_003859257.1 | 5661.XP_003859419.1 | 5661.XP_003859654.1 | 5661.XP_003859804.1 | 5661.XP_003860054.1 |
| 5661.XP_003859264.1 | 5661.XP_003859429.1 | 5661.XP_003859655.1 | 5661.XP_003859812.1 | 5661.XP_003860062.1 |
| 5661.XP_003859268.1 | 5661.XP_003859450.1 | 5661.XP_003859656.1 | 5661.XP_003859827.1 | 5661.XP_003860065.1 |
| 5661.XP_003859287.1 | 5661.XP_003859457.1 | 5661.XP_003859666.1 | 5661.XP_003859828.1 | 5661.XP_003860076.1 |
| 5661.XP_003859294.1 | 5661.XP_003859463.1 | 5661.XP_003859670.1 | 5661.XP_003859830.1 | 5661.XP_003860078.1 |
| 5661.XP_003859298.1 | 5661.XP_003859464.1 | 5661.XP_003859672.1 | 5661.XP_003859832.1 | 5661.XP_003860083.1 |
| 5661.XP_003859304.1 | 5661.XP_003859465.1 | 5661.XP_003859673.1 | 5661.XP_003859848.1 | 5661.XP_003860087.1 |
| 5661.XP_003859305.1 | 5661.XP_003859470.1 | 5661.XP_003859676.1 | 5661.XP_003859854.1 | 5661.XP_003860095.1 |
| 5661.XP_003859307.1 | 5661.XP_003859481.1 | 5661.XP_003859677.1 | 5661.XP_003859868.1 | 5661.XP_003860096.1 |
| 5661.XP_003859310.1 | 5661.XP_003859491.1 | 5661.XP_003859678.1 | 5661.XP_003859869.1 | 5661.XP_003860102.1 |
| 5661.XP_003859311.1 | 5661.XP_003859493.1 | 5661.XP_003859684.1 | 5661.XP_003859870.1 | 5661.XP_003860103.1 |
| 5661.XP_003859315.1 | 5661.XP_003859494.1 | 5661.XP_003859689.1 | 5661.XP_003859873.1 | 5661.XP_003860107.1 |
| 5661.XP_003859320.1 | 5661.XP_003859507.1 | 5661.XP_003859692.1 | 5661.XP_003859879.1 | 5661.XP_003860122.1 |
| 5661.XP_003859323.1 | 5661.XP_003859508.1 | 5661.XP_003859697.1 | 5661.XP_003859884.1 | 5661.XP_003860123.1 |
| 5661.XP_003859329.1 | 5661.XP_003859511.1 | 5661.XP_003859703.1 | 5661.XP_003859894.1 | 5661.XP_003860124.1 |
| 5661.XP_003859348.1 | 5661.XP_003859512.1 | 5661.XP_003859713.1 | 5661.XP_003859895.1 | 5661.XP_003860125.1 |
| 5661.XP_003859351.1 | 5661.XP_003859517.1 | 5661.XP_003859715.1 | 5661.XP_003859918.1 | 5661.XP_003860135.1 |
| 5661.XP_003859353.1 | 5661.XP_003859528.1 | 5661.XP_003859718.1 | 5661.XP_003859939.1 | 5661.XP_003860149.1 |
| 5661.XP_003859364.1 | 5661.XP_003859529.1 | 5661.XP_003859720.1 | 5661.XP_003859941.1 | 5661.XP_003860150.1 |
| 5661.XP_003859367.1 | 5661.XP_003859530.1 | 5661.XP_003859731.1 | 5661.XP_003859942.1 | 5661.XP_003860161.1 |
| 5661.XP_003860199.1 | 5661.XP_003859531.1 | 5661.XP_003859741.1 | 5661.XP_003859955.1 | 5661.XP_003860165.1 |
| 5661.XP_003860200.1 | 5661.XP_003859534.1 | 5661.XP_003859742.1 | 5661.XP_003859980.1 | 5661.XP_003860171.1 |
| 5661.XP_003860214.1 | 5661.XP_003859538.1 | 5661.XP_003859743.1 | 5661.XP_003859981.1 | 5661.XP_003860174.1 |
| 5661.XP_003860215.1 | 5661.XP_003859542.1 | 5661.XP_003859956.1 | 5661.XP_003859989.1 | 5661.XP_003860186.1 |
| 5661.XP_003860220.1 | 5661.XP_003859562.1 | 5661.XP_003859957.1 | 5661.XP_003859990.1 | 5661.XP_003860188.1 |
| 5661.XP_003860222.1 | 5661.XP_003859569.1 | 5661.XP_003859959.1 | 5661.XP_003859994.1 | 5661.XP_003860194.1 |
| 5661.XP_003860232.1 | 5661.XP_003859577.1 | 5661.XP_003859960.1 | 5661.XP_003860000.1 | 5661.XP_003860197.1 |
| 5661.XP_003860237.1 | 5661.XP_003859604.1 | 5661.XP_003859978.1 | 5661.XP_003860002.1 | 5661.XP_003860198.1 |
| 5661.XP_003860243.1 | 5661.XP_003860422.1 | 5661.XP_003860647.1 | 5661.XP_003860858.1 | 5661.XP_003861004.1 |
| 5661.XP_003860244.1 | 5661.XP_003860428.1 | 5661.XP_003860648.1 | 5661.XP_003860865.1 | 5661.XP_003861005.1 |
| 5661.XP_003860245.1 | 5661.XP_003860439.1 | 5661.XP_003860649.1 | 5661.XP_003860867.1 | 5661.XP_003861009.1 |
| 5661.XP_003860249.1 | 5661.XP_003860440.1 | 5661.XP_003860650.1 | 5661.XP_003860869.1 | 5661.XP_003861021.1 |
| 5661.XP_003860251.1 | 5661.XP_003860455.1 | 5661.XP_003860660.1 | 5661.XP_003860873.1 | 5661.XP_003861037.1 |
| 5661.XP_003860254.1 | 5661.XP_003860456.1 | 5661.XP_003860662.1 | 5661.XP_003860874.1 | 5661.XP_003861038.1 |
| 5661.XP_003860258.1 | 5661.XP_003860499.1 | 5661.XP_003860666.1 | 5661.XP_003860876.1 | 5661.XP_003861039.1 |
| 5661.XP_003860268.1 | 5661.XP_003860504.1 | 5661.XP_003860675.1 | 5661.XP_003860880.1 | 5661.XP_003861046.1 |
| 5661.XP_003860270.1 | 5661.XP_003860508.1 | 5661.XP_003860684.1 | 5661.XP_003860881.1 | 5661.XP_003861052.1 |
| 5661.XP_003860273.1 | 5661.XP_003860527.1 | 5661.XP_003860686.1 | 5661.XP_003860884.1 | 5661.XP_003861054.1 |
| 5661.XP_003860277.1 | 5661.XP_003860535.1 | 5661.XP_003860699.1 | 5661.XP_003860885.1 | 5661.XP_003861057.1 |
| 5661.XP_003860286.1 | 5661.XP_003860538.1 | 5661.XP_003860704.1 | 5661.XP_003860889.1 | 5661.XP_003861060.1 |
| 5661.XP_003860295.1 | 5661.XP_003860547.1 | 5661.XP_003860718.1 | 5661.XP_003860891.1 | 5661.XP_003861080.1 |
| 5661.XP_003860315.1 | 5661.XP_003860557.1 | 5661.XP_003860719.1 | 5661.XP_003860896.1 | 5661.XP_003861087.1 |
| 5661.XP_003860319.1 | 5661.XP_003860565.1 | 5661.XP_003860724.1 | 5661.XP_003860905.1 | 5661.XP_003861091.1 |
| 5661.XP_003860324.1 | 5661.XP_003860570.1 | 5661.XP_003860726.1 | 5661.XP_003860906.1 | 5661.XP_003861094.1 |
| 5661.XP_003860328.1 | 5661.XP_003860572.1 | 5661.XP_003860730.1 | 5661.XP_003860907.1 | 5661.XP_003861103.1 |
| 5661.XP_003860332.1 | 5661.XP_003860578.1 | 5661.XP_003860731.1 | 5661.XP_003860909.1 | 5661.XP_003861107.1 |
| 5661.XP_003860335.1 | 5661.XP_003860584.1 | 5661.XP_003860732.1 | 5661.XP_003860913.1 | 5661.XP_003861110.1 |
| 5661.XP_003860337.1 | 5661.XP_003860585.1 | 5661.XP_003860733.1 | 5661.XP_003860915.1 | 5661.XP_003861112.1 |
| 5661.XP_003860338.1 | 5661.XP_003860586.1 | 5661.XP_003860735.1 | 5661.XP_003860916.1 | 5661.XP_003861113.1 |
| 5661.XP_003860343.1 | 5661.XP_003860587.1 | 5661.XP_003860742.1 | 5661.XP_003860923.1 | 5661.XP_003861114.1 |
| 5661.XP_003860347.1 | 5661.XP_003860589.1 | 5661.XP_003860747.1 | 5661.XP_003860924.1 | 5661.XP_003861116.1 |
| 5661.XP_003860352.1 | 5661.XP_003860590.1 | 5661.XP_003860750.1 | 5661.XP_003860930.1 | 5661.XP_003861121.1 |
| 5661.XP_003860353.1 | 5661.XP_003860591.1 | 5661.XP_003860759.1 | 5661.XP_003860931.1 | 5661.XP_003861122.1 |
| 5661.XP_003860367.1 | 5661.XP_003860594.1 | 5661.XP_003860760.1 | 5661.XP_003860932.1 | 5661.XP_003861126.1 |
| 5661.XP_003860368.1 | 5661.XP_003860594.1 | 5661.XP_003860772.1 | 5661.XP_003860935.1 | 5661.XP_003861127.1 |
| 5661.XP_003860371.1 | 5661.XP_003860597.1 | 5661.XP_003860777.1 | 5661.XP_003860942.1 | 5661.XP_003861143.1 |
| 5661.XP_003860389.1 | 5661.XP_003860601.1 | 5661.XP_003860781.1 | 5661.XP_003860944.1 | 5661.XP_003861159.1 |
| 5661.XP_003860402.1 | 5661.XP_003860605.1 | 5661.XP_003860783.1 | 5661.XP_003860948.1 | 5661.XP_003861164.1 |
| 5661.XP_003860403.1 | 5661.XP_003860606.1 | 5661.XP_003860785.1 | 5661.XP_003860954.1 | 5661.XP_003861168.1 |
| 5661.XP_003860411.1 | 5661.XP_003860613.1 | 5661.XP_003860794.1 | 5661.XP_003860958.1 | 5661.XP_003861169.1 |
| 5661.XP_003861232.1 | 5661.XP_003860615.1 | 5661.XP_003860799.1 | 5661.XP_003860959.1 | 5661.XP_003861177.1 |
| 5661.XP_003861237.1 | 5661.XP_003860616.1 | 5661.XP_003860809.1 | 5661.XP_003860961.1 | 5661.XP_003861182.1 |
| 5661.XP_003861242.1 | 5661.XP_003860617.1 | 5661.XP_003860810.1 | 5661.XP_003860963.1 | 5661.XP_003861192.1 |
| 5661.XP_003861246.1 | 5661.XP_003860624.1 | 5661.XP_003860813.1 | 5661.XP_003860964.1 | 5661.XP_003861201.1 |
| 5661.XP_003861247.1 | 5661.XP_003860627.1 | 5661.XP_003860825.1 | 5661.XP_003860972.1 | 5661.XP_003861203.1 |
| 5661.XP_003861250.1 | 5661.XP_003860629.1 | 5661.XP_003860843.1 | 5661.XP_003860979.1 | 5661.XP_003861209.1 |
| 5661.XP_003861260.1 | 5661.XP_003860636.1 | 5661.XP_003860846.1 | 5661.XP_003860984.1 | 5661.XP_003861215.1 |
| 5661.XP_003861270.1 | 5661.XP_003860646.1 | 5661.XP_003860853.1 | 5661.XP_003860998.1 | 5661.XP_003861229.1 |
| 5661.XP_003861276.1 | 5661.XP_003861446.1 | 5661.XP_003861672.1 | 5661.XP_003861887.1 | 5661.XP_003862076.1 |
| 5661.XP_003861278.1 | 5661.XP_003861451.1 | 5661.XP_003861673.1 | 5661.XP_003861894.1 | 5661.XP_003862077.1 |
| 5661.XP_003861279.1 | 5661.XP_003861452.1 | 5661.XP_003861674.1 | 5661.XP_003861896.1 | 5661.XP_003862080.1 |
| 5661.XP_003861287.1 | 5661.XP_003861453.1 | 5661.XP_003861678.1 | 5661.XP_003861899.1 | 5661.XP_003862086.1 |
| 5661.XP_003861299.1 | 5661.XP_003861466.1 | 5661.XP_003861681.1 | 5661.XP_003861900.1 | 5661.XP_003862087.1 |
| 5661.XP_003861303.1 | 5661.XP_003861476.1 | 5661.XP_003861688.1 | 5661.XP_003861903.1 | 5661.XP_003862090.1 |
| 5661.XP_003861304.1 | 5661.XP_003861481.1 | 5661.XP_003861696.1 | 5661.XP_003861913.1 | 5661.XP_003862094.1 |
| 5661.XP_003861305.1 | 5661.XP_003861483.1 | 5661.XP_003861701.1 | 5661.XP_003861914.1 | 5661.XP_003862100.1 |
| 5661.XP_003861306.1 | 5661.XP_003861486.1 | 5661.XP_003861705.1 | 5661.XP_003861930.1 | 5661.XP_003862101.1 |
| 5661.XP_003861314.1 | 5661.XP_003861488.1 | 5661.XP_003861706.1 | 5661.XP_003861940.1 | 5661.XP_003862104.1 |
| 5661.XP_003861323.1 | 5661.XP_003861492.1 | 5661.XP_003861709.1 | 5661.XP_003861942.1 | 5661.XP_003862110.1 |
| 5661.XP_003861325.1 | 5661.XP_003861504.1 | 5661.XP_003861710.1 | 5661.XP_003861948.1 | 5661.XP_003862113.1 |
| 5661.XP_003861329.1 | 5661.XP_003861509.1 | 5661.XP_003861712.1 | 5661.XP_003861949.1 | 5661.XP_003862116.1 |
| 5661.XP_003861333.1 | 5661.XP_003861544.1 | 5661.XP_003861719.1 | 5661.XP_003861950.1 | 5661.XP_003862121.1 |
| 5661.XP_003861343.1 | 5661.XP_003861546.1 | 5661.XP_003861720.1 | 5661.XP_003861961.1 | 5661.XP_003862125.1 |
| 5661.XP_003861344.1 | 5661.XP_003861549.1 | 5661.XP_003861722.1 | 5661.XP_003861964.1 | 5661.XP_003862126.1 |
| 5661.XP_003861348.1 | 5661.XP_003861552.1 | 5661.XP_003861726.1 | 5661.XP_003861967.1 | 5661.XP_003862127.1 |
| 5661.XP_003861361.1 | 5661.XP_003861575.1 | 5661.XP_003861740.1 | 5661.XP_003861968.1 | 5661.XP_003862130.1 |
| 5661.XP_003861374.1 | 5661.XP_003861577.1 | 5661.XP_003861745.1 | 5661.XP_003861976.1 | 5661.XP_003862132.1 |
| 5661.XP_003861376.1 | 5661.XP_003861578.1 | 5661.XP_003861746.1 | 5661.XP_003861983.1 | 5661.XP_003862140.1 |
| 5661.XP_003861384.1 | 5661.XP_003861586.1 | 5661.XP_003861747.1 | 5661.XP_003861989.1 | 5661.XP_003862146.1 |
| 5661.XP_003861393.1 | 5661.XP_003861590.1 | 5661.XP_003861748.1 | 5661.XP_003861990.1 | 5661.XP_003862149.1 |
| 5661.XP_003861404.1 | 5661.XP_003861591.1 | 5661.XP_003861765.1 | 5661.XP_003861998.1 | 5661.XP_003862155.1 |
| 5661.XP_003861405.1 | 5661.XP_003861592.1 | 5661.XP_003861777.1 | 5661.XP_003862002.1 | 5661.XP_003862156.1 |
| 5661.XP_003861408.1 | 5661.XP_003861601.1 | 5661.XP_003861795.1 | 5661.XP_003862005.1 | 5661.XP_003862157.1 |
| 5661.XP_003861413.1 | 5661.XP_003861605.1 | 5661.XP_003861808.1 | 5661.XP_003862010.1 | 5661.XP_003862172.1 |
| 5661.XP_003861420.1 | 5661.XP_003861609.1 | 5661.XP_003861810.1 | 5661.XP_003862013.1 | 5661.XP_003862176.1 |
| 5661.XP_003861424.1 | 5661.XP_003861615.1 | 5661.XP_003861814.1 | 5661.XP_003862014.1 | 5661.XP_003862186.1 |
| 5661.XP_003861434.1 | 5661.XP_003861618.1 | 5661.XP_003861820.1 | 5661.XP_003862021.1 | 5661.XP_003862189.1 |
| 5661.XP_003861436.1 | 5661.XP_003861619.1 | 5661.XP_003861821.1 | 5661.XP_003862024.1 | 5661.XP_003862197.1 |
| 5661.XP_003861443.1 | 5661.XP_003861620.1 | 5661.XP_003861826.1 | 5661.XP_003862030.1 | 5661.XP_003862204.1 |
| 5661.XP_003861444.1 | 5661.XP_003861621.1 | 5661.XP_003861835.1 | 5661.XP_003862032.1 | 5661.XP_003862210.1 |
| 5661.XP_003862241.1 | 5661.XP_003861625.1 | 5661.XP_003861840.1 | 5661.XP_003862041.1 | 5661.XP_003862211.1 |
| 5661.XP_003862242.1 | 5661.XP_003861630.1 | 5661.XP_003861844.1 | 5661.XP_003862045.1 | 5661.XP_003862220.1 |
| 5661.XP_003862244.1 | 5661.XP_003861631.1 | 5661.XP_003861854.1 | 5661.XP_003862046.1 | 5661.XP_003862224.1 |
| 5661.XP_003862245.1 | 5661.XP_003861649.1 | 5661.XP_003861857.1 | 5661.XP_003862048.1 | 5661.XP_003862225.1 |
| 5661.XP_003862246.1 | 5661.XP_003861651.1 | 5661.XP_003861858.1 | 5661.XP_003862063.1 | 5661.XP_003862230.1 |
| 5661.XP_003862247.1 | 5661.XP_003861665.1 | 5661.XP_003861862.1 | 5661.XP_003862065.1 | 5661.XP_003862234.1 |
| 5661.XP_003862248.1 | 5661.XP_003861666.1 | 5661.XP_003861863.1 | 5661.XP_003862068.1 | 5661.XP_003862237.1 |
| 5661.XP_003862252.1 | 5661.XP_003861671.1 | 5661.XP_003861883.1 | 5661.XP_003862073.1 | 5661.XP_003862238.1 |
| 5661.XP_003862253.1 | 5661.XP_003862370.1 | 5661.XP_003862561.1 | 5661.XP_003862736.1 | 5661.XP_003862954.1 |
| 5661.XP_003862257.1 | 5661.XP_003862373.1 | 5661.XP_003862566.1 | 5661.XP_003862740.1 | 5661.XP_003862959.1 |
| 5661.XP_003862265.1 | 5661.XP_003862374.1 | 5661.XP_003862568.1 | 5661.XP_003862752.1 | 5661.XP_003862960.1 |
| 5661.XP_003862269.1 | 5661.XP_003862376.1 | 5661.XP_003862570.1 | 5661.XP_003862754.1 | 5661.XP_003862964.1 |
| 5661.XP_003862272.1 | 5661.XP_003862382.1 | 5661.XP_003862574.1 | 5661.XP_003862756.1 | 5661.XP_003862965.1 |
| 5661.XP_003862274.1 | 5661.XP_003862384.1 | 5661.XP_003862587.1 | 5661.XP_003862760.1 | 5661.XP_003862970.1 |
| 5661.XP_003862279.1 | 5661.XP_003862385.1 | 5661.XP_003862597.1 | 5661.XP_003862761.1 | 5661.XP_003862971.1 |
| 5661.XP_003862285.1 | 5661.XP_003862392.1 | 5661.XP_003862600.1 | 5661.XP_003862763.1 | 5661.XP_003862973.1 |
| 5661.XP_003862289.1 | 5661.XP_003862394.1 | 5661.XP_003862602.1 | 5661.XP_003862768.1 | 5661.XP_003862985.1 |
| 5661.XP_003862290.1 | 5661.XP_003862396.1 | 5661.XP_003862608.1 | 5661.XP_003862770.1 | 5661.XP_003862989.1 |
| 5661.XP_003862293.1 | 5661.XP_003862400.1 | 5661.XP_003862610.1 | 5661.XP_003862774.1 | 5661.XP_003862994.1 |
| 5661.XP_003862295.1 | 5661.XP_003862403.1 | 5661.XP_003862613.1 | 5661.XP_003862777.1 | 5661.XP_003863003.1 |
| 5661.XP_003862299.1 | 5661.XP_003862408.1 | 5661.XP_003862626.1 | 5661.XP_003862778.1 | 5661.XP_003863010.1 |
| 5661.XP_003862308.1 | 5661.XP_003862417.1 | 5661.XP_003862632.1 | 5661.XP_003862779.1 | 5661.XP_003863011.1 |
| 5661.XP_003862309.1 | 5661.XP_003862418.1 | 5661.XP_003862636.1 | 5661.XP_003862789.1 | 5661.XP_003863014.1 |
| 5661.XP_003862313.1 | 5661.XP_003862421.1 | 5661.XP_003862638.1 | 5661.XP_003862790.1 | 5661.XP_003863016.1 |
| 5661.XP_003862318.1 | 5661.XP_003862424.1 | 5661.XP_003862639.1 | 5661.XP_003862793.1 | 5661.XP_003863019.1 |
| 5661.XP_003862319.1 | 5661.XP_003862430.1 | 5661.XP_003862644.1 | 5661.XP_003862798.1 | 5661.XP_003863022.1 |
| 5661.XP_003862320.1 | 5661.XP_003862432.1 | 5661.XP_003862645.1 | 5661.XP_003862807.1 | 5661.XP_003863023.1 |
| 5661.XP_003862321.1 | 5661.XP_003862442.1 | 5661.XP_003862649.1 | 5661.XP_003862808.1 | 5661.XP_003863028.1 |
| 5661.XP_003862324.1 | 5661.XP_003862443.1 | 5661.XP_003862653.1 | 5661.XP_003862814.1 | 5661.XP_003863034.1 |
| 5661.XP_003862328.1 | 5661.XP_003862446.1 | 5661.XP_003862660.1 | 5661.XP_003862822.1 | 5661.XP_003863035.1 |
| 5661.XP_003862335.1 | 5661.XP_003862463.1 | 5661.XP_003862661.1 | 5661.XP_003862849.1 | 5661.XP_003863040.1 |
| 5661.XP_003862343.1 | 5661.XP_003862481.1 | 5661.XP_003862665.1 | 5661.XP_003862850.1 | 5661.XP_003863041.1 |
| 5661.XP_003862346.1 | 5661.XP_003862492.1 | 5661.XP_003862674.1 | 5661.XP_003862855.1 | 5661.XP_003863042.1 |
| 5661.XP_003862351.1 | 5661.XP_003862495.1 | 5661.XP_003862678.1 | 5661.XP_003862869.1 | 5661.XP_003863044.1 |
| 5661.XP_003862355.1 | 5661.XP_003862496.1 | 5661.XP_003862680.1 | 5661.XP_003862870.1 | 5661.XP_003863046.1 |
| 5661.XP_003862358.1 | 5661.XP_003862510.1 | 5661.XP_003862683.1 | 5661.XP_003862882.1 | 5661.XP_003863050.1 |
| 5661.XP_003862359.1 | 5661.XP_003862514.1 | 5661.XP_003862684.1 | 5661.XP_003862888.1 | 5661.XP_003863054.1 |
| 5661.XP_003862363.1 | 5661.XP_003862515.1 | 5661.XP_003862686.1 | 5661.XP_003862890.1 | 5661.XP_003863055.1 |
| 5661.XP_003862364.1 | 5661.XP_003862517.1 | 5661.XP_003862695.1 | 5661.XP_003862891.1 | 5661.XP_003863060.1 |
| 5661.XP_003862368.1 | 5661.XP_003862518.1 | 5661.XP_003862696.1 | 5661.XP_003862892.1 | 5661.XP_003863063.1 |
| 5661.XP_003863101.1 | 5661.XP_003862527.1 | 5661.XP_003862697.1 | 5661.XP_003862900.1 | 5661.XP_003863067.1 |
| 5661.XP_003863107.1 | 5661.XP_003862533.1 | 5661.XP_003862709.1 | 5661.XP_003862906.1 | 5661.XP_003863076.1 |
| 5661.XP_003863110.1 | 5661.XP_003862534.1 | 5661.XP_003862712.1 | 5661.XP_003862922.1 | 5661.XP_003863082.1 |
| 5661.XP_003863111.1 | 5661.XP_003862535.1 | 5661.XP_003862718.1 | 5661.XP_003862927.1 | 5661.XP_003863083.1 |
| 5661.XP_003863112.1 | 5661.XP_003862536.1 | 5661.XP_003862724.1 | 5661.XP_003862938.1 | 5661.XP_003863088.1 |
| 5661.XP_003863116.1 | 5661.XP_003862550.1 | 5661.XP_003862728.1 | 5661.XP_003862939.1 | 5661.XP_003863092.1 |
| 5661.XP_003863126.1 | 5661.XP_003862553.1 | 5661.XP_003862729.1 | 5661.XP_003862942.1 | 5661.XP_003863093.1 |
| 5661.XP_003863127.1 | 5661.XP_003862558.1 | 5661.XP_003862733.1 | 5661.XP_003862948.1 | 5661.XP_003863096.1 |
| 5661.XP_003863129.1 | 5661.XP_003863383.1 | 5661.XP_003863652.1 | 5661.XP_003863859.1 | 5661.XP_003864086.1 |
| 5661.XP_003863143.1 | 5661.XP_003863390.1 | 5661.XP_003863663.1 | 5661.XP_003863860.1 | 5661.XP_003864089.1 |
| 5661.XP_003863148.1 | 5661.XP_003863392.1 | 5661.XP_003863664.1 | 5661.XP_003863861.1 | 5661.XP_003864092.1 |
| 5661.XP_003863149.1 | 5661.XP_003863393.1 | 5661.XP_003863667.1 | 5661.XP_003863865.1 | 5661.XP_003864094.1 |
| 5661.XP_003863150.1 | 5661.XP_003863401.1 | 5661.XP_003863671.1 | 5661.XP_003863866.1 | 5661.XP_003864098.1 |
| 5661.XP_003863151.1 | 5661.XP_003863405.1 | 5661.XP_003863682.1 | 5661.XP_003863872.1 | 5661.XP_003864099.1 |
| 5661.XP_003863158.1 | 5661.XP_003863406.1 | 5661.XP_003863690.1 | 5661.XP_003863877.1 | 5661.XP_003864103.1 |
| 5661.XP_003863163.1 | 5661.XP_003863414.1 | 5661.XP_003863691.1 | 5661.XP_003863882.1 | 5661.XP_003864110.1 |
| 5661.XP_003863182.1 | 5661.XP_003863446.1 | 5661.XP_003863692.1 | 5661.XP_003863899.1 | 5661.XP_003864112.1 |
| 5661.XP_003863199.1 | 5661.XP_003863460.1 | 5661.XP_003863693.1 | 5661.XP_003863911.1 | 5661.XP_003864119.1 |
| 5661.XP_003863215.1 | 5661.XP_003863464.1 | 5661.XP_003863696.1 | 5661.XP_003863918.1 | 5661.XP_003864144.1 |
| 5661.XP_003863220.1 | 5661.XP_003863465.1 | 5661.XP_003863698.1 | 5661.XP_003863920.1 | 5661.XP_003864148.1 |
| 5661.XP_003863221.1 | 5661.XP_003863478.1 | 5661.XP_003863706.1 | 5661.XP_003863925.1 | 5661.XP_003864153.1 |
| 5661.XP_003863225.1 | 5661.XP_003863484.1 | 5661.XP_003863708.1 | 5661.XP_003863936.1 | 5661.XP_003864157.1 |
| 5661.XP_003863237.1 | 5661.XP_003863490.1 | 5661.XP_003863709.1 | 5661.XP_003863939.1 | 5661.XP_003864169.1 |
| 5661.XP_003863255.1 | 5661.XP_003863509.1 | 5661.XP_003863714.1 | 5661.XP_003863963.1 | 5661.XP_003864172.1 |
| 5661.XP_003863268.1 | 5661.XP_003863512.1 | 5661.XP_003863723.1 | 5661.XP_003863967.1 | 5661.XP_003864173.1 |
| 5661.XP_003863282.1 | 5661.XP_003863521.1 | 5661.XP_003863726.1 | 5661.XP_003863969.1 | 5661.XP_003864177.1 |
| 5661.XP_003863283.1 | 5661.XP_003863522.1 | 5661.XP_003863733.1 | 5661.XP_003863977.1 | 5661.XP_003864178.1 |
| 5661.XP_003863286.1 | 5661.XP_003863525.1 | 5661.XP_003863734.1 | 5661.XP_003863982.1 | 5661.XP_003864179.1 |
| 5661.XP_003863295.1 | 5661.XP_003863556.1 | 5661.XP_003863742.1 | 5661.XP_003863989.1 | 5661.XP_003864180.1 |
| 5661.XP_003863309.1 | 5661.XP_003863562.1 | 5661.XP_003863743.1 | 5661.XP_003863992.1 | 5661.XP_003864181.1 |
| 5661.XP_003863320.1 | 5661.XP_003863568.1 | 5661.XP_003863745.1 | 5661.XP_003863997.1 | 5661.XP_003864182.1 |
| 5661.XP_003863323.1 | 5661.XP_003863573.1 | 5661.XP_003863757.1 | 5661.XP_003863999.1 | 5661.XP_003864188.1 |
| 5661.XP_003863328.1 | 5661.XP_003863591.1 | 5661.XP_003863758.1 | 5661.XP_003864003.1 | 5661.XP_003864194.1 |
| 5661.XP_003863329.1 | 5661.XP_003863593.1 | 5661.XP_003863761.1 | 5661.XP_003864004.1 | 5661.XP_003864197.1 |
| 5661.XP_003863343.1 | 5661.XP_003863606.1 | 5661.XP_003863762.1 | 5661.XP_003864005.1 | 5661.XP_003864198.1 |
| 5661.XP_003863355.1 | 5661.XP_003863607.1 | 5661.XP_003863763.1 | 5661.XP_003864007.1 | 5661.XP_003864200.1 |
| 5661.XP_003863357.1 | 5661.XP_003863608.1 | 5661.XP_003863768.1 | 5661.XP_003864011.1 | 5661.XP_003864203.1 |
| 5661.XP_003863358.1 | 5661.XP_003863609.1 | 5661.XP_003863769.1 | 5661.XP_003864018.1 | 5661.XP_003864211.1 |
| 5661.XP_003863359.1 | 5661.XP_003863610.1 | 5661.XP_003863777.1 | 5661.XP_003864022.1 | 5661.XP_003864216.1 |
| 5661.XP_003863374.1 | 5661.XP_003863620.1 | 5661.XP_003863791.1 | 5661.XP_003864027.1 | 5661.XP_003864224.1 |
| 5661.XP_003864254.1 | 5661.XP_003863621.1 | 5661.XP_003863793.1 | 5661.XP_003864029.1 | 5661.XP_003864234.1 |
| 5661.XP_003864257.1 | 5661.XP_003863630.1 | 5661.XP_003863794.1 | 5661.XP_003864035.1 | 5661.XP_003864242.1 |
| 5661.XP_003864268.1 | 5661.XP_003863634.1 | 5661.XP_003863802.1 | 5661.XP_003864046.1 | 5661.XP_003864243.1 |
| 5661.XP_003864271.1 | 5661.XP_003863635.1 | 5661.XP_003863830.1 | 5661.XP_003864068.1 | 5661.XP_003864247.1 |
| 5661.XP_003864273.1 | 5661.XP_003863636.1 | 5661.XP_003863838.1 | 5661.XP_003864070.1 | 5661.XP_003864248.1 |
| 5661.XP_003864275.1 | 5661.XP_003863642.1 | 5661.XP_003863849.1 | 5661.XP_003864076.1 | 5661.XP_003864249.1 |
| 5661.XP_003864279.1 | 5661.XP_003863648.1 | 5661.XP_003863854.1 | 5661.XP_003864077.1 | 5661.XP_003864251.1 |
| 5661.XP_003864284.1 | 5661.XP_003863651.1 | 5661.XP_003863858.1 | 5661.XP_003864078.1 | 5661.XP_003864253.1 |
| 5661.XP_003864291.1 | 5661.XP_003864486.1 | 5661.XP_003864705.1 | 5661.XP_003864873.1 | 5661.XP_003865010.1 |
| 5661.XP_003864299.1 | 5661.XP_003864487.1 | 5661.XP_003864706.1 | 5661.XP_003864878.1 | 5661.XP_003865017.1 |
| 5661.XP_003864306.1 | 5661.XP_003864489.1 | 5661.XP_003864709.1 | 5661.XP_003864882.1 | 5661.XP_003865021.1 |
| 5661.XP_003864340.1 | 5661.XP_003864493.1 | 5661.XP_003864712.1 | 5661.XP_003864885.1 | 5661.XP_003865026.1 |
| 5661.XP_003864344.1 | 5661.XP_003864497.1 | 5661.XP_003864719.1 | 5661.XP_003864890.1 | 5661.XP_003865043.1 |
| 5661.XP_003864346.1 | 5661.XP_003864499.1 | 5661.XP_003864724.1 | 5661.XP_003864892.1 | 5661.XP_003865055.1 |
| 5661.XP_003864348.1 | 5661.XP_003864503.1 | 5661.XP_003864727.1 | 5661.XP_003864893.1 | 5661.XP_003865058.1 |
| 5661.XP_003864350.1 | 5661.XP_003864506.1 | 5661.XP_003864729.1 | 5661.XP_003864894.1 | 5661.XP_003865060.1 |
| 5661.XP_003864356.1 | 5661.XP_003864512.1 | 5661.XP_003864730.1 | 5661.XP_003864900.1 | 5661.XP_003865063.1 |
| 5661.XP_003864362.1 | 5661.XP_003864517.1 | 5661.XP_003864734.1 | 5661.XP_003864905.1 | 5661.XP_003865068.1 |
| 5661.XP_003864370.1 | 5661.XP_003864525.1 | 5661.XP_003864739.1 | 5661.XP_003864908.1 | 5661.XP_003865071.1 |
| 5661.XP_003864377.1 | 5661.XP_003864530.1 | 5661.XP_003864742.1 | 5661.XP_003864910.1 | 5661.XP_003865072.1 |
| 5661.XP_003864378.1 | 5661.XP_003864534.1 | 5661.XP_003864751.1 | 5661.XP_003864913.1 | 5661.XP_003865093.1 |
| 5661.XP_003864379.1 | 5661.XP_003864540.1 | 5661.XP_003864754.1 | 5661.XP_003864914.1 | 5661.XP_003865095.1 |
| 5661.XP_003864383.1 | 5661.XP_003864548.1 | 5661.XP_003864756.1 | 5661.XP_003864919.1 | 5661.XP_003865097.1 |
| 5661.XP_003864390.1 | 5661.XP_003864570.1 | 5661.XP_003864757.1 | 5661.XP_003864920.1 | 5661.XP_003865099.1 |
| 5661.XP_003864391.1 | 5661.XP_003864571.1 | 5661.XP_003864768.1 | 5661.XP_003864921.1 | 5661.XP_003865105.1 |
| 5661.XP_003864400.1 | 5661.XP_003864576.1 | 5661.XP_003864770.1 | 5661.XP_003864932.1 | 5661.XP_003865109.1 |
| 5661.XP_003864403.1 | 5661.XP_003864593.1 | 5661.XP_003864771.1 | 5661.XP_003864936.1 | 5661.XP_003865117.1 |
| 5661.XP_003864405.1 | 5661.XP_003864595.1 | 5661.XP_003864772.1 | 5661.XP_003864939.1 | 5661.XP_003865122.1 |
| 5661.XP_003864409.1 | 5661.XP_003864596.1 | 5661.XP_003864780.1 | 5661.XP_003864944.1 | 5661.XP_003865127.1 |
| 5661.XP_003864417.1 | 5661.XP_003864598.1 | 5661.XP_003864782.1 | 5661.XP_003864946.1 | 5661.XP_003865129.1 |
| 5661.XP_003864421.1 | 5661.XP_003864617.1 | 5661.XP_003864785.1 | 5661.XP_003864947.1 | 5661.XP_003865134.1 |
| 5661.XP_003864427.1 | 5661.XP_003864625.1 | 5661.XP_003864789.1 | 5661.XP_003864953.1 | 5661.XP_003865137.1 |
| 5661.XP_003864430.1 | 5661.XP_003864629.1 | 5661.XP_003864798.1 | 5661.XP_003864961.1 | 5661.XP_003865138.1 |
| 5661.XP_003864432.1 | 5661.XP_003864633.1 | 5661.XP_003864799.1 | 5661.XP_003864963.1 | 5661.XP_003865141.1 |
| 5661.XP_003864440.1 | 5661.XP_003864634.1 | 5661.XP_003864800.1 | 5661.XP_003864968.1 | 5661.XP_003865142.1 |
| 5661.XP_003864456.1 | 5661.XP_003864641.1 | 5661.XP_003864802.1 | 5661.XP_003864969.1 | 5661.XP_003865144.1 |
| 5661.XP_003864463.1 | 5661.XP_003864648.1 | 5661.XP_003864803.1 | 5661.XP_003864970.1 | 5661.XP_003865152.1 |
| 5661.XP_003864473.1 | 5661.XP_003864652.1 | 5661.XP_003864811.1 | 5661.XP_003864971.1 | 5661.XP_003865161.1 |
| 5661.XP_003864475.1 | 5661.XP_003864653.1 | 5661.XP_003864816.1 | 5661.XP_003864978.1 | 5661.XP_003865166.1 |
| 5661.XP_003864480.1 | 5661.XP_003864658.1 | 5661.XP_003864819.1 | 5661.XP_003864979.1 | 5661.XP_003865169.1 |
| 5661.XP_003865204.1 | 5661.XP_003864666.1 | 5661.XP_003864826.1 | 5661.XP_003864983.1 | 5661.XP_003865172.1 |
| 5661.XP_003865205.1 | 5661.XP_003864670.1 | 5661.XP_003864828.1 | 5661.XP_003864984.1 | 5661.XP_003865176.1 |
| 5661.XP_003865206.1 | 5661.XP_003864671.1 | 5661.XP_003864838.1 | 5661.XP_003864985.1 | 5661.XP_003865178.1 |
| 5661.XP_003865209.1 | 5661.XP_003864685.1 | 5661.XP_003864839.1 | 5661.XP_003864986.1 | 5661.XP_003865179.1 |
| 5661.XP_003865210.1 | 5661.XP_003864690.1 | 5661.XP_003864850.1 | 5661.XP_003864987.1 | 5661.XP_003865182.1 |
| 5661.XP_003865212.1 | 5661.XP_003864695.1 | 5661.XP_003864857.1 | 5661.XP_003864989.1 | 5661.XP_003865183.1 |
| 5661.XP_003865213.1 | 5661.XP_003864703.1 | 5661.XP_003864858.1 | 5661.XP_003864995.1 | 5661.XP_003865186.1 |
| 5661.XP_003865216.1 | 5661.XP_003864704.1 | 5661.XP_003864868.1 | 5661.XP_003865000.1 | 5661.XP_003865189.1 |
| 5661.XP_003865217.1 | 5661.XP_003865341.1 | 5661.XP_003865487.1 | 5661.XP_003865687.1 |  |
| 5661.XP_003865221.1 | 5661.XP_003865342.1 | 5661.XP_003865489.1 | 5661.XP_003865688.1 |  |
| 5661.XP_003865222.1 | 5661.XP_003865345.1 | 5661.XP_003865498.1 | 5661.XP_003865689.1 |  |
| 5661.XP_003865223.1 | 5661.XP_003865353.1 | 5661.XP_003865507.1 | 5661.XP_003865690.1 |  |
| 5661.XP_003865236.1 | 5661.XP_003865354.1 | 5661.XP_003865508.1 | 5661.XP_003865693.1 |  |
| 5661.XP_003865247.1 | 5661.XP_003865356.1 | 5661.XP_003865509.1 | 5661.XP_003865699.1 |  |
| 5661.XP_003865255.1 | 5661.XP_003865357.1 | 5661.XP_003865510.1 | 5661.XP_003865704.1 |  |
| 5661.XP_003865256.1 | 5661.XP_003865362.1 | 5661.XP_003865513.1 | 5661.XP_003865712.1 |  |
| 5661.XP_003865257.1 | 5661.XP_003865363.1 | 5661.XP_003865514.1 | 5661.XP_003865715.1 |  |
| 5661.XP_003865258.1 | 5661.XP_003865364.1 | 5661.XP_003865517.1 | 5661.XP_003865729.1 |  |
| 5661.XP_003865264.1 | 5661.XP_003865370.1 | 5661.XP_003865521.1 | 5661.XP_003865734.1 |  |
| 5661.XP_003865273.1 | 5661.XP_003865381.1 | 5661.XP_003865524.1 | 5661.XP_003865744.1 |  |
| 5661.XP_003865274.1 | 5661.XP_003865386.1 | 5661.XP_003865553.1 | 5661.XP_003865747.1 |  |
| 5661.XP_003865283.1 | 5661.XP_003865389.1 | 5661.XP_003865556.1 | 5661.XP_003865750.1 |  |
| 5661.XP_003865286.1 | 5661.XP_003865393.1 | 5661.XP_003865560.1 | 5661.XP_003865765.1 |  |
| 5661.XP_003865287.1 | 5661.XP_003865394.1 | 5661.XP_003865571.1 | 5661.XP_003865825.1 |  |
| 5661.XP_003865289.1 | 5661.XP_003865400.1 | 5661.XP_003865576.1 |  |  |
| 5661.XP_003865299.1 | 5661.XP_003865402.1 | 5661.XP_003865579.1 |  |  |
| 5661.XP_003865302.1 | 5661.XP_003865404.1 | 5661.XP_003865581.1 |  |  |
| 5661.XP_003865305.1 | 5661.XP_003865413.1 | 5661.XP_003865583.1 |  |  |
| 5661.XP_003865307.1 | 5661.XP_003865417.1 | 5661.XP_003865584.1 |  |  |
| 5661.XP_003865308.1 | 5661.XP_003865419.1 | 5661.XP_003865585.1 |  |  |
| 5661.XP_003865311.1 | 5661.XP_003865425.1 | 5661.XP_003865587.1 |  |  |
| 5661.XP_003865317.1 | 5661.XP_003865429.1 | 5661.XP_003865589.1 |  |  |
| 5661.XP_003865320.1 | 5661.XP_003865430.1 | 5661.XP_003865600.1 |  |  |
| 5661.XP_003865322.1 | 5661.XP_003865431.1 | 5661.XP_003865602.1 |  |  |
| 5661.XP_003865324.1 | 5661.XP_003865432.1 | 5661.XP_003865608.1 |  |  |
| 5661.XP_003865328.1 | 5661.XP_003865433.1 | 5661.XP_003865609.1 |  |  |
| 5661.XP_003865329.1 | 5661.XP_003865437.1 | 5661.XP_003865619.1 |  |  |
| 5661.XP_003865332.1 | 5661.XP_003865440.1 | 5661.XP_003865626.1 |  |  |
| 5661.XP_003865334.1 | 5661.XP_003865443.1 | 5661.XP_003865630.1 |  |  |
| 5661.XP_003865338.1 | 5661.XP_003865447.1 | 5661.XP_003865638.1 |  |  |
| 5661.XP_003865448.1 | 5661.XP_003865643.1 | 5661.XP_003865769.1 |  |  |
| 5661.XP_003865449.1 | 5661.XP_003865646.1 | 5661.XP_003865771.1 |  |  |
| 5661.XP_003865450.1 | 5661.XP_003865649.1 | 5661.XP_003865778.1 |  |  |
| 5661.XP_003865451.1 | 5661.XP_003865655.1 | 5661.XP_003865779.1 |  |  |
| 5661.XP_003865458.1 | 5661.XP_003865672.1 | 5661.XP_003865792.1 |  |  |
| 5661.XP_003865472.1 | 5661.XP_003865675.1 | 5661.XP_003865797.1 |  |  |
| 5661.XP_003865478.1 | 5661.XP_003865676.1 | 5661.XP_003865807.1 |  |  |
| 5661.XP_003865486.1 | 5661.XP_003865677.1 | 5661.XP_003865813.1 |  |  |
